# Supplementary material for: Free circular introns with an unusual branchpoint in neuronal projections
Source: eLife. 2019 Nov 7;8:e47809. doi: 10.7554/eLife.47809 (PMC6879206; doi:10.7554/eLife.47809)
Supplement: Source code 1. [file elife-47809-code1.gz › Saini19-supplement/code_for_saini_et_al_19/Rscript_plots_Saini_et_al_2019.nb.html]

Code to replot figures for Saini et al. 2019


Code 

- Show All Code
- Hide All Code
- Download Rmd

# Code to replot figures for Saini et al. 2019

#### *Harleen Saini*

#### *October 19, 2019*


```
colnames(cbp5rep_kal)
```


```
 [1] "ens_geneid"  "ext_geneid"  "target_id"   "pval"        "qval"        "b"           "se_b"        "mean_obs"   
 [9] "cbr1_tpm"    "cbr2_tpm"    "cbr3_tpm"    "cbr4_tpm"    "cbr5_tpm"    "pr1_tpm"     "pr2_tpm"     "pr3_tpm"    
[17] "pr4_tpm"     "pr5_tpm"     "mean_cb_tpm" "mean_p_tpm"
```


```
colnames(intron_master)
```


```
 [1] "chr"                  "start"                "end"                  "strand"               "intron_id"           
 [6] "ens_geneid"           "ext_geneid"           "intron_len"           "cbr1_intron"          "cbr2_intron"         
[11] "cbr3_intron"          "cbr4_intron"          "cbr5_intron"          "pr1_intron"           "pr2_intron"          
[16] "pr3_intron"           "pr4_intron"           "pr5_intron"           "polya_intron"         "ribo_intron"         
[21] "cbr1_ee"              "cbr2_ee"              "cbr3_ee"              "cbr4_ee"              "cbr5_ee"             
[26] "pr1_ee"               "pr2_ee"               "pr3_ee"               "pr4_ee"               "pr5_ee"              
[31] "polya_ee"             "ribo_ee"              "cbr1_ei"              "cbr2_ei"              "cbr3_ei"             
[36] "cbr4_ei"              "cbr5_ei"              "pr1_ei"               "pr2_ei"               "pr3_ei"              
[41] "pr4_ei"               "pr5_ei"               "polya_ei"             "ribo_ei"              "cbr1_ie"             
[46] "cbr2_ie"              "cbr3_ie"              "cbr4_ie"              "cbr5_ie"              "pr1_ie"              
[51] "pr2_ie"               "pr3_ie"               "pr4_ie"               "pr5_ie"               "polya_ie"            
[56] "ribo_ie"              "CBpas_intron"         "Ppas_intron"          "circ_num_cb"          "circ_num_p"          
[61] "mean_cb_intron"       "mean_p_intron"        "cb_intron_perlen"     "p_intron_perlen"      "mean_cb_ee"          
[66] "mean_p_ee"            "mean_cb_ei"           "mean_p_ei"            "mean_cb_ie"           "mean_p_ie"           
[71] "P_over_CB_intron_log" "cb_thresh"            "p_thresh"
```


PhyloP scores across regions of interest:


```
p_free_deplet_phyloP <- read.delim("../data_tables_for_saini_et_al_19/Intermediate_tables_to_reproduce_figures/phyloP_free_introns_depleted_from_projections.tsv", header=T, stringsAsFactors=F)
p_free_enrich_phyloP <- read.delim("../data_tables_for_saini_et_al_19/Intermediate_tables_to_reproduce_figures/phyloP_free_introns_enriched_in_projections.tsv", header=T, stringsAsFactors=F)
p_all_retained_phyloP <- read.delim("../data_tables_for_saini_et_al_19/Intermediate_tables_to_reproduce_figures/phyloP_retained_introns_in_projections.tsv", header=T, stringsAsFactors=F)
all_exons_phyloP <- read.delim("../data_tables_for_saini_et_al_19/Intermediate_tables_to_reproduce_figures/phyloP_all_exons.tsv", header=T, stringsAsFactors=F)
p_retained_enrich_deplet_exons_phyloP <- rbind(p_all_retained_phyloP, p_free_enrich_phyloP, p_free_deplet_phyloP, all_exons_phyloP)
```


Data for GO term enrichment analysis:


```
# For GO term analysis
p_enriched <- cbp5rep_kal %>% filter(qval < 0.01 & b > 0 & mean_obs > 1) %>% select(ext_geneid, ens_geneid)
cb_enriched <- cbp5rep_kal %>% filter(qval < 0.01 & b < 0 & mean_obs > 1) %>% select(ext_geneid, ens_geneid)
background_genes <- cbp5rep_kal %>% filter(mean_obs > 1) %>% select(ext_geneid, ens_geneid)
```


Setting up manually selected mRNA/introns to label on plots


```
label_rp_mRNAs <- Go_5840_rp_rev$V1
cbp5rep_kal$label_rp_mRNAs <- NA
cbp5rep_kal$label_rp_mRNAs <- cbp5rep_kal$ens_gene %in% label_rp_mRNAs
label_diffexp_rna <- c("ENSRNOT00000092445.1", "ENSRNOT00000080218.1", "ENSRNOT00000034401.5", "ENSRNOT00000068493.2", "ENSRNOT00000013461.7",
                       "ENSRNOT00000068013.3","ENSRNOT00000085692.1", "ENSRNOT00000022828.7", "ENSRNOT00000007583.7",
                       "ENSRNOT00000078535.1", "ENSRNOT00000080943.1", "ENSRNOT00000090095.1", "ENSRNOT11111111111.1","ENSRNOT00000093096.1")
cbp5rep_kal$label_diffexp_rna <- cbp5rep_kal$target_id %in% label_diffexp_rna
# To label Genes with CIRTs from Buckley et al. 2011  
label_cirts <- c("Adcy2", "Adcy3", "Adcy4", "Adcy5", "App", "Cacna1b", "Cacna1h", "Camk2b", "Camk2d", "Camk2g",
                            "Cdh1", "Creb1", "Fmr1", "Gabbr1", "Gabrd", "Gabra4", "Gabrg3", "Gria3","AABR07041374.1","Gria4",
                 "Grik1", "Grin1", "Grm7", "Kcnd2", "Map2", "Snca", "Stx1b" )
intron_master$label_cirts <- NA
intron_master$label_cirts <- intron_master$ext_geneid %in% label_cirts
```


```
# Subsetting introns  
p_introns_thresh <- intron_master %>% filter(pr1_intron >= 1 & pr2_intron >= 1 & pr3_intron >= 1 & pr4_intron >=1 & pr5_intron >= 1) %>%
       filter(p_intron_perlen > 0.005)
# 
cb_introns_thresh <- intron_master %>% filter(cbr1_intron >= 1 & cbr2_intron >= 1 & cbr3_intron >= 1 & cbr4_intron >=1 & cbr5_intron >= 1) %>%
       filter(cb_intron_perlen > 0.005)
# nrow(intron_master %>% filter(cb_thresh == T))  
# p = 1632  
# cb = 57432  
# common = 1599, only p = 33  
p_introns_EI <- p_introns_thresh %>% filter(mean_p_ei >= 1 & mean_p_ie < 1)
p_introns_IE <- p_introns_thresh %>% filter(mean_p_ie >= 1 & mean_p_ei < 1)
p_introns_free <- p_introns_thresh %>% filter(mean_p_ie < 1 & mean_p_ei < 1)
p_introns_retained <- p_introns_thresh %>% filter(mean_p_ie >= 1 & mean_p_ei >= 1)
p_introns_free_noPA_noRibo <- p_introns_free %>% filter(Ppas_intron == 0 & ribo_intron < 15)
#nrow(p_introns_free %>% filter(Ppas_intron > 0)) # 96  
#nrow(p_introns_free %>% filter(Ppas_intron == 0 & ribo_intron >= 15)) # 221  
p_introns_free_noPA_noRibo_enrich <- p_introns_free_noPA_noRibo %>% filter(P_over_CB_intron_log >= 0)
p_introns_free_noPA_noRibo_deplet <- p_introns_free_noPA_noRibo %>% filter(P_over_CB_intron_log < 0)
cb_introns_EI <- cb_introns_thresh %>% filter(mean_cb_ei >= 1 & mean_cb_ie < 1)
cb_introns_IE <- cb_introns_thresh %>% filter(mean_cb_ie >= 1 & mean_cb_ei < 1)
cb_introns_free <- cb_introns_thresh %>% filter(mean_cb_ie < 1 & mean_cb_ei < 1)
cb_introns_retained <- cb_introns_thresh %>% filter(mean_cb_ie >= 1 & mean_cb_ei >= 1)
cb_introns_free_noPA_noRibo <- cb_introns_free %>% filter(CBpas_intron == 0 & ribo_intron < 15)
# Combining intron table with kallisto/sleuth output  
intron_master_cbp_kal <- left_join(intron_master, cbp5rep_kal, by = c("ens_geneid","ext_geneid"))
p_introns_free_noPA_noRibo$introntype <- as.factor("free")
p_introns_retained$introntype <- as.factor("retained")
p_introns_free_retained <- bind_rows(p_introns_free_noPA_noRibo, p_introns_retained)
p_introns_free_retained_cbp_kal <- left_join(p_introns_free_retained, cbp5rep_kal, by = c("ens_geneid","ext_geneid"))
```


~~~~ PLOTS ~~~


```
fig2 <- ggplot(cbp5rep_kal, aes(mean_obs, b)) +
  geom_point(alpha = 0.05, size=1, color="gray30", shape = 16) + 
  geom_vline(xintercept=3, linetype="dotted", size=0.1) + 
  geom_hline(yintercept=0, linetype="dotted", size=0.1) + 
  geom_point(data=cbp5rep_kal[cbp5rep_kal$qval < 0.01 & cbp5rep_kal$b >0,], color= "#FF6532", alpha = 0.4, size =1, shape = 16) +
  geom_point(data=cbp5rep_kal[cbp5rep_kal$qval < 0.01 & cbp5rep_kal$b <0,], color= "#0066CC", alpha = 0.4, size =1, shape = 16) +
  geom_point(data=cbp5rep_kal[cbp5rep_kal$label_rp_mRNAs == TRUE & cbp5rep_kal$qval < 0.01 & cbp5rep_kal$mean_obs > 3,], color= "black", alpha = 1, size =1.0) +
  geom_point(data=cbp5rep_kal[cbp5rep_kal$label_rp_mRNAs == TRUE & cbp5rep_kal$qval < 0.01 & cbp5rep_kal$mean_obs > 3,], color= "black", alpha = 1, size =1.0) +
  geom_point(data=cbp5rep_kal[cbp5rep_kal$label_diffexp_rna == TRUE,], shape=23, size = 2.5, color= "black", stroke=0.4) +
  geom_label_repel(data=cbp5rep_kal[cbp5rep_kal$label_diffexp_rna == TRUE,], aes(label=ext_geneid),color= "white", size=3, fill = "gray47", min.segment.length = 0, segment.colour = "black") +
  ylim(-5,5) + scale_x_continuous(position = "top") +
  xlab("Mean ln(est. read counts)") + ylab("ln(projections/whole-cells fold difference)") +
  theme(panel.grid.major = element_blank(), panel.grid.minor = element_blank(), panel.background = element_blank(), axis.line = element_line(colour = 'black', size = 0.2))
fig2
```


```
fig3 <- ggplot(intron_master, aes((mean_cb_intron+1), (mean_p_intron+1))) +
  geom_point(data=intron_master[intron_master$cb_thresh == F & intron_master$p_thresh == F,], color="gray30", alpha = 0.1) + 
  geom_abline(linetype="dashed", size=0.2) + 
  geom_point(data=intron_master[intron_master$cb_thresh == T,], color="#0066CC", alpha = 0.5) +
  geom_point(data=intron_master[intron_master$p_thresh == T,], color="#FF6532", alpha = 0.5) +
  geom_point(data=intron_master[intron_master$intron_id == "ENSRNOG00000016770_001i",], shape=23, size = 3, color= "black", stroke=1) +
  geom_point(data=intron_master[intron_master$intron_id == "ENSRNOG00000005513_005i",], shape=23, size = 3, color= "black", stroke=1) +       
  geom_label_repel(data=intron_master[intron_master$intron_id == "ENSRNOG00000005513_005i" | intron_master$intron_id == "ENSRNOG00000016770_001i",], aes(label=ext_geneid), color= "white", size=3, fill = "gray47", alpha=0.9, box.padding = 0.25, point.padding = 0.1) +
  scale_x_log10(limits=c(1,10^5), breaks = trans_breaks("log10", function(x) 10^x), labels = trans_format("log10", math_format(10^.x))) +
  scale_y_log10(limits=c(1,10^5), breaks = trans_breaks("log10", function(x) 10^x), labels = trans_format("log10", math_format(10^.x))) +
  xlab("Mean read counts (Whole cells)") + ylab("Mean read counts (Projections)") +
  theme(panel.grid.major = element_blank(), panel.grid.minor = element_blank(), panel.background = element_blank(), axis.line = element_line(colour = "black", size=0.2)) 
fig3
```


```
fig3_figsupp2 <- ggplot(intron_master, aes((mean_cb_intron+1), (mean_p_intron+1))) +
  geom_point(data=intron_master[intron_master$cb_thresh == F & intron_master$p_thresh == F,], color="gray30", alpha = 0.1) + 
  geom_abline(linetype="dashed", size=0.2) + 
  geom_point(data=intron_master[intron_master$cb_thresh == T,], color="#0066CC", alpha = 0.5) +
  geom_point(data=intron_master[intron_master$p_thresh == T,], color="#FF6532", alpha = 0.5) +
  geom_point(data=intron_master[intron_master$label_cirts == T ,], color="black") +
  geom_label_repel(data=intron_master[intron_master$label_cirts == T & intron_master$p_thresh == T, ], aes(label=ext_geneid), color= "white", size=3, fill = "gray47", min.segment.length = 0, segment.colour = "black") +
  scale_x_log10(limits=c(1,10^5), breaks = trans_breaks("log10", function(x) 10^x), labels = trans_format("log10", math_format(10^.x))) +
  scale_y_log10(limits=c(1,10^5), breaks = trans_breaks("log10", function(x) 10^x), labels = trans_format("log10", math_format(10^.x))) +
  xlab("Mean read counts (Whole cells)") + ylab("Mean read counts (Projections)") +
  theme(panel.grid.major = element_blank(), panel.grid.minor = element_blank(), panel.background = element_blank(), axis.line = element_line(colour = "black", size=0.2)) 
fig3_figsupp2
```


```
fig4 <- ggplot(p_introns_thresh, aes(mean_p_ie+0.1, mean_p_ei+0.1)) +
  geom_point(alpha = 0.7, color="gray30") + 
  geom_hline(yintercept=1, linetype="dashed", size = 0.5) + 
  geom_vline(xintercept=1, linetype="dashed", size=0.5) + 
  geom_point(data=p_introns_thresh[p_introns_thresh$intron_id  == "ENSRNOG00000016770_001i",], shape=23, size = 3, color= "black", stroke=1) + #Calm2
  geom_point(data=p_introns_thresh[p_introns_thresh$intron_id  == "ENSRNOG00000007686_010i",], shape=23, size = 3, color= "black", stroke=1) + # Sept3
  geom_point(data=p_introns_thresh[p_introns_thresh$intron_id  == "ENSRNOG00000006235_022i",], shape=23, size = 3, color= "black", stroke=1) + # Nell2
  geom_point(data=p_introns_thresh[p_introns_thresh$intron_id  == "ENSRNOG00000017500_005i",], shape=23, size = 3, color= "black", stroke=1) + # Mtss1l
  geom_point(data=p_introns_thresh[p_introns_thresh$intron_id  == "ENSRNOG00000001557_007i",], shape=23, size = 3, color= "black", stroke=1) +
  geom_point(data=p_introns_thresh[p_introns_thresh$intron_id  == "ENSRNOG00000020851_005i",], shape=23, size = 3, color= "black", stroke=1) +
  geom_point(data=p_introns_thresh[p_introns_thresh$intron_id  == "ENSRNOG00000055300_029i",], shape=23, size = 3, color= "black", stroke=1) + # Ncapd2  
  geom_label_repel(data=p_introns_thresh[p_introns_thresh$intron_id == "ENSRNOG00000016770_001i", ], aes(label=ext_geneid),  color= "white", size=3, fill = "gray47", min.segment.length = 0, segment.colour = "black") +
  geom_label_repel(data=p_introns_thresh[p_introns_thresh$intron_id == "ENSRNOG00000007686_010i", ], aes(label=ext_geneid),  color= "white", size=3, fill = "gray47", min.segment.length = 0, segment.colour = "black") +
  geom_label_repel(data=p_introns_thresh[p_introns_thresh$intron_id == "ENSRNOG00000006235_022i", ], aes(label=ext_geneid),  color= "white", size=3, fill = "gray47", min.segment.length = 0, segment.colour = "black") +
  geom_label_repel(data=p_introns_thresh[p_introns_thresh$intron_id == "ENSRNOG00000017500_005i", ], aes(label=ext_geneid),  color= "white", size=3, fill = "gray47", min.segment.length = 0, segment.colour = "black") +
  geom_label_repel(data=p_introns_thresh[p_introns_thresh$intron_id == "ENSRNOG00000001557_007i", ], aes(label=ext_geneid),  color= "white", size=3, fill = "gray47", min.segment.length = 0, segment.colour = "black") +
  geom_label_repel(data=p_introns_thresh[p_introns_thresh$intron_id == "ENSRNOG00000020851_005i", ], aes(label=ext_geneid),  color= "white", size=3, fill = "gray47", min.segment.length = 0, segment.colour = "black") +
  geom_label_repel(data=p_introns_thresh[p_introns_thresh$intron_id == "ENSRNOG00000055300_029i", ], aes(label=ext_geneid),  color= "white", size=3, fill = "gray47", min.segment.length = 0, segment.colour = "black") +
  scale_x_log10(limits=c(0.1,30000)) + scale_y_log10(limits=c(0.1,30000)) +
  xlab("Mean intron-exon reads") + ylab("Mean exon-intron reads") +
  theme(panel.grid.major = element_blank(), panel.grid.minor = element_blank(), panel.background = element_blank(), axis.line = element_line(colour = "black", size=0.2))
fig4
```


```
fig5 <- ggplot(p_introns_free_noPA_noRibo, aes((cb_intron_perlen+p_intron_perlen)/2, P_over_CB_intron_log)) +
  geom_point(alpha = 0.7, color="gray30", size = 1) + geom_hline(yintercept= 0, linetype="dashed", size=0.2) +
  geom_point(data = p_introns_free_noPA_noRibo[p_introns_free_noPA_noRibo$circ_num_p >= 2 & p_introns_free_noPA_noRibo$P_over_CB_intron_log > 0,], alpha = 1, color="red", size = 1) +
  geom_point(data = p_introns_free_noPA_noRibo[p_introns_free_noPA_noRibo$intron_id == "ENSRNOG00000003207_027i" | p_introns_free_noPA_noRibo$intron_id == "ENSRNOG00000057078_001i" | p_introns_free_noPA_noRibo$intron_id == "ENSRNOG00000007622_006i",], alpha = 1, color="red", size = 1) +
  geom_label_repel(data = p_introns_free_noPA_noRibo[p_introns_free_noPA_noRibo$circ_num_p >= 2 & p_introns_free_noPA_noRibo$P_over_CB_intron_log > 0,], aes(label=ext_geneid), color= "white", size=3, fill = "gray47", min.segment.length = 0, segment.colour = "black") +
  geom_label_repel(data = p_introns_free_noPA_noRibo[p_introns_free_noPA_noRibo$intron_id == "ENSRNOG00000003207_027i" | p_introns_free_noPA_noRibo$intron_id == "ENSRNOG00000057078_001i" | p_introns_free_noPA_noRibo$intron_id == "ENSRNOG00000007622_006i",], aes(label=ext_geneid), color= "white", size=3, fill = "gray47", min.segment.length = 0, segment.colour = "black") +
  geom_label_repel(data = p_introns_free_noPA_noRibo[p_introns_free_noPA_noRibo$intron_id == "ENSRNOG00000005513_005i" | p_introns_free_noPA_noRibo$intron_id == "ENSRNOG00000025768_010i" | p_introns_free_noPA_noRibo$intron_id == "ENSRNOG00000055300_029i" | p_introns_free_noPA_noRibo$intron_id == "ENSRNOG00000010768_005i" | p_introns_free_noPA_noRibo$intron_id == "ENSRNOG00000009722_012i" | p_introns_free_noPA_noRibo$intron_id == "ENSRNOG00000012999_007i",], aes(label=ext_geneid), color= "white", size=3, fill = "gray47", min.segment.length = 0, segment.colour = "black") +
  scale_x_log10() + ylim(-5,5) +
  xlab("Mean read density per intron") + ylab("ln (projections/whole-cells) read counts") +
  theme(panel.grid.major = element_blank(), panel.grid.minor = element_blank(), panel.background = element_blank(), axis.line = element_line(colour = "black", size=0.2))
fig5
```


```
fig6c <- ggplot(p_retained_enrich_deplet_exons_phyloP, aes(x=median_cons, color=sample)) + stat_ecdf() +
  xlim(-0.5,4) +
  xlab("Median phyloP score") + ylab("Fraction of data") +
  theme(panel.grid.major = element_blank(), panel.grid.minor = element_blank(), panel.background = element_blank(), axis.line = element_line(colour = "black", size=0.2)) 
fig6c
```


```
fig4_figsupp2A <- ggplot(p_introns_free_retained_cbp_kal[p_introns_free_retained_cbp_kal$introntype == "free",], aes(b, P_over_CB_intron_log)) +
  geom_point(alpha = 0.5, size=0.5, color="gray30") + 
  geom_hline(yintercept=0, linetype="dashed", size=0.2) + geom_vline(xintercept= 0, linetype="dashed", size=0.2) +
  geom_point(data = p_introns_free_retained_cbp_kal[p_introns_free_retained_cbp_kal$introntype == "free" & p_introns_free_retained_cbp_kal$circ_num_p >= 2 & p_introns_free_retained_cbp_kal$P_over_CB_intron_log > 0,], alpha = 1, color="red", size = 1) +
  geom_point(data = p_introns_free_retained_cbp_kal[p_introns_free_retained_cbp_kal$introntype == "free" & p_introns_free_retained_cbp_kal$intron_id == "ENSRNOG00000003207_027i" | p_introns_free_retained_cbp_kal$intron_id == "ENSRNOG00000057078_001i" | p_introns_free_retained_cbp_kal$intron_id == "ENSRNOG00000007622_006i",], alpha = 1, color="red", size = 1) +
  xlim(-5,5) + ylim(-5,5) +
  xlab("ln (projections/whole-cells) mRNA fold difference") + ylab("ln (projections/whole-cells) reads per intron region") +
  ggtitle("Free intron versus mRNA enrichment in projections") +
  theme(panel.grid.major = element_blank(), panel.grid.minor = element_blank(), panel.background = element_blank(), axis.line = element_line(colour = "black", size=0.2))
fig4_figsupp2A
```


```
fig4_figsupp2B <- ggplot(p_introns_free_retained_cbp_kal[p_introns_free_retained_cbp_kal$introntype == "retained",], aes(b, P_over_CB_intron_log)) +
  geom_point(alpha = 0.5, size=0.5, color="gray30") + 
  geom_hline(yintercept=0, linetype="dashed", size=0.2) + geom_vline(xintercept= 0, linetype="dashed", size=0.2) +
  geom_point(data=p_introns_free_retained_cbp_kal[p_introns_free_retained_cbp_kal$introntype == "retained" & p_introns_free_retained_cbp_kal$ext_geneid == "Sept3",], size = 1, color= "black") +
  geom_point(data=p_introns_free_retained_cbp_kal[p_introns_free_retained_cbp_kal$introntype == "retained" & p_introns_free_retained_cbp_kal$intron_id == "ENSRNOG00000016770_001i",], size = 1, color= "black") +
  geom_label_repel(data = p_introns_free_retained_cbp_kal[p_introns_free_retained_cbp_kal$introntype == "retained" & p_introns_free_retained_cbp_kal$intron_id == "ENSRNOG00000016770_001i" | p_introns_free_retained_cbp_kal$ext_geneid == "Sept3",], aes(label=ext_geneid), color= "white", size=3, fill = "gray47") +
  xlim(-5,5) + ylim(-5,5) +
  xlab("ln (projections/whole-cells) mRNA fold difference") + ylab("ln (projections/whole-cells) reads per intron region") +
  ggtitle("Retained intron versus mRNA enrichment in projections") +
  theme(panel.grid.major = element_blank(), panel.grid.minor = element_blank(), panel.background = element_blank(), axis.line = element_line(colour = "black", size=0.2))
fig4_figsupp2B
```


```
#nrow(p_introns_free_retained_cbp_kal %>% filter(introntype == "retained") %>%  
#       filter(P_over_CB_intron_log > 0 & b <= 0))  
#nrow(p_introns_free_retained_cbp_kal %>% filter(introntype == "free") %>%  
#       filter(P_over_CB_intron_log <= 0 & b > 0))  
# upper right = 120 (retained) and 31 (free)  
# upper left = 6 and 22  
# lower right = 280 and 146 
# lower left = 232 and 183
```


```
fig2_figsupp1 <- pheatmap(corr_mat_cbp5rep, scale = "none",
         fontsize_row = 8, 
         fontsize_col = 8,
         cellwidth=20,
         cellheight = 20,
         color = colorRampPalette(brewer.pal(n=8, name = "YlOrRd"))(50),
         cluster_cols=F,
         cluster_rows=F,
         clustering_distance_cols = "euclidean", 
         clustering_method = "complete",
         annotation_legend = TRUE, 
         display_numbers= T)
fig2_figsupp1
```


```
sessionInfo()
```


```
R version 3.5.1 (2018-07-02)
Platform: x86_64-apple-darwin15.6.0 (64-bit)
Running under: macOS High Sierra 10.13.6

Matrix products: default
BLAS: /System/Library/Frameworks/Accelerate.framework/Versions/A/Frameworks/vecLib.framework/Versions/A/libBLAS.dylib
LAPACK: /Library/Frameworks/R.framework/Versions/3.5/Resources/lib/libRlapack.dylib

locale:
[1] en_US.UTF-8/en_US.UTF-8/en_US.UTF-8/C/en_US.UTF-8/en_US.UTF-8

attached base packages:
[1] stats     graphics  grDevices utils     datasets  methods   base     

other attached packages:
[1] bindrcpp_0.2.2     pheatmap_1.0.12    tidyr_0.8.2        RColorBrewer_1.1-2 ggrepel_0.8.0      scales_1.0.0      
[7] ggplot2_3.1.0      dplyr_0.7.8        reshape2_1.4.3    

loaded via a namespace (and not attached):
 [1] Rcpp_1.0.0       pillar_1.3.1     compiler_3.5.1   plyr_1.8.4       bindr_0.1.1      base64enc_0.1-3 
 [7] tools_3.5.1      digest_0.6.18    jsonlite_1.6     evaluate_0.12    tibble_2.0.1     gtable_0.2.0    
[13] pkgconfig_2.0.2  rlang_0.3.1      rstudioapi_0.9.0 yaml_2.2.0       xfun_0.4         withr_2.1.2     
[19] stringr_1.3.1    knitr_1.21       grid_3.5.1       tidyselect_0.2.5 glue_1.3.0       R6_2.3.0        
[25] rmarkdown_1.11   purrr_0.3.0      magrittr_1.5     htmltools_0.3.6  assertthat_0.2.0 colorspace_1.4-0
[31] labeling_0.3     stringi_1.2.4    lazyeval_0.2.1   munsell_0.5.0    crayon_1.3.4
```


LS0tCnRpdGxlOiAiQ29kZSB0byByZXBsb3QgZmlndXJlcyBmb3IgU2FpbmkgZXQgYWwuIDIwMTkiCmF1dGhvcjogSGFybGVlbiBTYWluaQpkYXRlOiBPY3RvYmVyIDE5LCAyMDE5Cm91dHB1dDogaHRtbF9ub3RlYm9vawotLS0KCmBgYHtyLCBlY2hvPUZBTFNFLCByZXN1bHRzPSdoaWRlJywgZmlnLmtlZXA9J2FsbCd9CiMgTG9hZGluZyBMaWJyYXJpZXMKbGlicmFyeShyZXNoYXBlMikKbGlicmFyeShkcGx5cikKbGlicmFyeShnZ3Bsb3QyKQpsaWJyYXJ5KHNjYWxlcykKbGlicmFyeShnZ3JlcGVsKQpsaWJyYXJ5KFJDb2xvckJyZXdlcikKbGlicmFyeSh0aWR5cikKbGlicmFyeShwaGVhdG1hcCkKYGBgCgpgYGB7ciwgZWNobz1GQUxTRX0KIyBSZWFkaW5nIGRhdGEgZmlsZXMgIApjYnA1cmVwX2thbCA8LSByZWFkLmRlbGltKCIuLi9kYXRhX3RhYmxlc19mb3Jfc2FpbmlfZXRfYWxfMTkvRmlndXJlMi1Tb3VyY2VEYXRhMV9STkFfZGlmZmV4cF9XQ192c19QLnRzdiIsIGhlYWRlcj1ULCBzZXA9Ilx0IiwgY29tbWVudC5jaGFyID0gJyMnKQppbnRyb25fbWFzdGVyIDwtIHJlYWQuZGVsaW0oIi4uL2RhdGFfdGFibGVzX2Zvcl9zYWluaV9ldF9hbF8xOS9GaWd1cmUzLVNvdXJjZURhdGExX0FsbF9JbnRyb25zLnRzdiIsIGhlYWRlcj1ULCBzZXA9Ilx0IiwgY29tbWVudC5jaGFyID0gJyMnICkKCiMgVG8gZ2V0IG5hbWVzIG9mIHJpYm9zb21hbCBwcm90ZWlucywgZG93bmxvYWRlZCBnZW5lcyBvZiBnZW5lIG9udG9sb2d5IGNhdGVnb3J5IEdPXzAwMDU4NDAgYW5kIG1hbnVhbGx5IHNlbGVjdGVkIGFsbCBSUEwgYW5kIFJQUyBnZW5lcyAoZXhsdWRlZCBraW5hc2VzKS4gIApHb181ODQwX3JwX3JldiA8LSByZWFkLmRlbGltKCIuLi9kYXRhX3RhYmxlc19mb3Jfc2FpbmlfZXRfYWxfMTkvR09fMDAwNTg0MF9yaWJvc29tYWxfcHJvdGVpbl9JRHMudHN2IiwgaGVhZGVyPUYpCnNhbXBsZXMgPC0gcmVhZC5jc3YoIi4uL2RhdGFfdGFibGVzX2Zvcl9zYWluaV9ldF9hbF8xOS9jYnA1cmVwX2NvcnJfc2FtcGxlcy5jc3YiLCBoZWFkZXI9VCkKYGBgCgpgYGB7cn0KY29sbmFtZXMoY2JwNXJlcF9rYWwpCmBgYAoKYGBge3J9CmNvbG5hbWVzKGludHJvbl9tYXN0ZXIpCmBgYAoKUGh5bG9QIHNjb3JlcyBhY3Jvc3MgcmVnaW9ucyBvZiBpbnRlcmVzdDoKYGBge3J9CnBfZnJlZV9kZXBsZXRfcGh5bG9QIDwtIHJlYWQuZGVsaW0oIi4uL2RhdGFfdGFibGVzX2Zvcl9zYWluaV9ldF9hbF8xOS9waHlsb1BfZnJlZV9pbnRyb25zX2RlcGxldGVkX2Zyb21fcHJvamVjdGlvbnMudHN2IiwgaGVhZGVyPVQsIHN0cmluZ3NBc0ZhY3RvcnM9RikKcF9mcmVlX2VucmljaF9waHlsb1AgPC0gcmVhZC5kZWxpbSgiLi4vZGF0YV90YWJsZXNfZm9yX3NhaW5pX2V0X2FsXzE5L3BoeWxvUF9mcmVlX2ludHJvbnNfZW5yaWNoZWRfaW5fcHJvamVjdGlvbnMudHN2IiwgaGVhZGVyPVQsIHN0cmluZ3NBc0ZhY3RvcnM9RikKcF9hbGxfcmV0YWluZWRfcGh5bG9QIDwtIHJlYWQuZGVsaW0oIi4uL2RhdGFfdGFibGVzX2Zvcl9zYWluaV9ldF9hbF8xOS9waHlsb1BfcmV0YWluZWRfaW50cm9uc19pbl9wcm9qZWN0aW9ucy50c3YiLCBoZWFkZXI9VCwgc3RyaW5nc0FzRmFjdG9ycz1GKQphbGxfZXhvbnNfcGh5bG9QIDwtIHJlYWQuZGVsaW0oIi4uL2RhdGFfdGFibGVzX2Zvcl9zYWluaV9ldF9hbF8xOS9waHlsb1BfYWxsX2V4b25zLnRzdiIsIGhlYWRlcj1ULCBzdHJpbmdzQXNGYWN0b3JzPUYpCgpwX3JldGFpbmVkX2VucmljaF9kZXBsZXRfZXhvbnNfcGh5bG9QIDwtIHJiaW5kKHBfYWxsX3JldGFpbmVkX3BoeWxvUCwgcF9mcmVlX2VucmljaF9waHlsb1AsIHBfZnJlZV9kZXBsZXRfcGh5bG9QLCBhbGxfZXhvbnNfcGh5bG9QKQpgYGAKCkRhdGEgZm9yIEdPIHRlcm0gZW5yaWNobWVudCBhbmFseXNpczoKYGBge3J9CiMgRm9yIEdPIHRlcm0gYW5hbHlzaXMKcF9lbnJpY2hlZCA8LSBjYnA1cmVwX2thbCAlPiUgZmlsdGVyKHF2YWwgPCAwLjAxICYgYiA+IDAgJiBtZWFuX29icyA+IDEpICU+JSBzZWxlY3QoZXh0X2dlbmVpZCwgZW5zX2dlbmVpZCkKY2JfZW5yaWNoZWQgPC0gY2JwNXJlcF9rYWwgJT4lIGZpbHRlcihxdmFsIDwgMC4wMSAmIGIgPCAwICYgbWVhbl9vYnMgPiAxKSAlPiUgc2VsZWN0KGV4dF9nZW5laWQsIGVuc19nZW5laWQpCmJhY2tncm91bmRfZ2VuZXMgPC0gY2JwNXJlcF9rYWwgJT4lIGZpbHRlcihtZWFuX29icyA+IDEpICU+JSBzZWxlY3QoZXh0X2dlbmVpZCwgZW5zX2dlbmVpZCkKYGBgCgpTZXR0aW5nIHVwIG1hbnVhbGx5IHNlbGVjdGVkIG1STkEvaW50cm9ucyB0byBsYWJlbCBvbiBwbG90cyAgCmBgYHtyfQpsYWJlbF9ycF9tUk5BcyA8LSBHb181ODQwX3JwX3JldiRWMQpjYnA1cmVwX2thbCRsYWJlbF9ycF9tUk5BcyA8LSBOQQpjYnA1cmVwX2thbCRsYWJlbF9ycF9tUk5BcyA8LSBjYnA1cmVwX2thbCRlbnNfZ2VuZSAlaW4lIGxhYmVsX3JwX21STkFzCgpsYWJlbF9kaWZmZXhwX3JuYSA8LSBjKCJFTlNSTk9UMDAwMDAwOTI0NDUuMSIsICJFTlNSTk9UMDAwMDAwODAyMTguMSIsICJFTlNSTk9UMDAwMDAwMzQ0MDEuNSIsICJFTlNSTk9UMDAwMDAwNjg0OTMuMiIsICJFTlNSTk9UMDAwMDAwMTM0NjEuNyIsCiAgICAgICAgICAgICAgICAgICAgICAgIkVOU1JOT1QwMDAwMDA2ODAxMy4zIiwiRU5TUk5PVDAwMDAwMDg1NjkyLjEiLCAiRU5TUk5PVDAwMDAwMDIyODI4LjciLCAiRU5TUk5PVDAwMDAwMDA3NTgzLjciLAogICAgICAgICAgICAgICAgICAgICAgICJFTlNSTk9UMDAwMDAwNzg1MzUuMSIsICJFTlNSTk9UMDAwMDAwODA5NDMuMSIsICJFTlNSTk9UMDAwMDAwOTAwOTUuMSIsICJFTlNSTk9UMTExMTExMTExMTEuMSIsIkVOU1JOT1QwMDAwMDA5MzA5Ni4xIikKY2JwNXJlcF9rYWwkbGFiZWxfZGlmZmV4cF9ybmEgPC0gY2JwNXJlcF9rYWwkdGFyZ2V0X2lkICVpbiUgbGFiZWxfZGlmZmV4cF9ybmEKCiMgVG8gbGFiZWwgR2VuZXMgd2l0aCBDSVJUcyBmcm9tIEJ1Y2tsZXkgZXQgYWwuIDIwMTEgIApsYWJlbF9jaXJ0cyA8LSBjKCJBZGN5MiIsICJBZGN5MyIsICJBZGN5NCIsICJBZGN5NSIsICJBcHAiLCAiQ2FjbmExYiIsICJDYWNuYTFoIiwgIkNhbWsyYiIsICJDYW1rMmQiLCAiQ2FtazJnIiwKICAgICAgICAgICAgICAgICAgICAgICAgICAgICJDZGgxIiwgIkNyZWIxIiwgIkZtcjEiLCAiR2FiYnIxIiwgIkdhYnJkIiwgIkdhYnJhNCIsICJHYWJyZzMiLCAiR3JpYTMiLCJBQUJSMDcwNDEzNzQuMSIsIkdyaWE0IiwKICAgICAgICAgICAgICAgICAiR3JpazEiLCAiR3JpbjEiLCAiR3JtNyIsICJLY25kMiIsICJNYXAyIiwgIlNuY2EiLCAiU3R4MWIiICkKaW50cm9uX21hc3RlciRsYWJlbF9jaXJ0cyA8LSBOQQppbnRyb25fbWFzdGVyJGxhYmVsX2NpcnRzIDwtIGludHJvbl9tYXN0ZXIkZXh0X2dlbmVpZCAlaW4lIGxhYmVsX2NpcnRzCgpgYGAKCmBgYHtyLCByZXN1bHRzPSdoaWRlJywgd2FybmluZyA9IEZBTFNFfQojIFN1YnNldHRpbmcgaW50cm9ucyAgCnBfaW50cm9uc190aHJlc2ggPC0gaW50cm9uX21hc3RlciAlPiUgZmlsdGVyKHByMV9pbnRyb24gPj0gMSAmIHByMl9pbnRyb24gPj0gMSAmIHByM19pbnRyb24gPj0gMSAmIHByNF9pbnRyb24gPj0xICYgcHI1X2ludHJvbiA+PSAxKSAlPiUKICAgICAgIGZpbHRlcihwX2ludHJvbl9wZXJsZW4gPiAwLjAwNSkKIyAKY2JfaW50cm9uc190aHJlc2ggPC0gaW50cm9uX21hc3RlciAlPiUgZmlsdGVyKGNicjFfaW50cm9uID49IDEgJiBjYnIyX2ludHJvbiA+PSAxICYgY2JyM19pbnRyb24gPj0gMSAmIGNicjRfaW50cm9uID49MSAmIGNicjVfaW50cm9uID49IDEpICU+JQogICAgICAgZmlsdGVyKGNiX2ludHJvbl9wZXJsZW4gPiAwLjAwNSkKCiMgbnJvdyhpbnRyb25fbWFzdGVyICU+JSBmaWx0ZXIoY2JfdGhyZXNoID09IFQpKSAgCiMgcCA9IDE2MzIgIAojIGNiID0gNTc0MzIgIAojIGNvbW1vbiA9IDE1OTksIG9ubHkgcCA9IDMzICAKCnBfaW50cm9uc19FSSA8LSBwX2ludHJvbnNfdGhyZXNoICU+JSBmaWx0ZXIobWVhbl9wX2VpID49IDEgJiBtZWFuX3BfaWUgPCAxKQpwX2ludHJvbnNfSUUgPC0gcF9pbnRyb25zX3RocmVzaCAlPiUgZmlsdGVyKG1lYW5fcF9pZSA+PSAxICYgbWVhbl9wX2VpIDwgMSkKcF9pbnRyb25zX2ZyZWUgPC0gcF9pbnRyb25zX3RocmVzaCAlPiUgZmlsdGVyKG1lYW5fcF9pZSA8IDEgJiBtZWFuX3BfZWkgPCAxKQpwX2ludHJvbnNfcmV0YWluZWQgPC0gcF9pbnRyb25zX3RocmVzaCAlPiUgZmlsdGVyKG1lYW5fcF9pZSA+PSAxICYgbWVhbl9wX2VpID49IDEpCnBfaW50cm9uc19mcmVlX25vUEFfbm9SaWJvIDwtIHBfaW50cm9uc19mcmVlICU+JSBmaWx0ZXIoUHBhc19pbnRyb24gPT0gMCAmIHJpYm9faW50cm9uIDwgMTUpCgojbnJvdyhwX2ludHJvbnNfZnJlZSAlPiUgZmlsdGVyKFBwYXNfaW50cm9uID4gMCkpICMgOTYgIAojbnJvdyhwX2ludHJvbnNfZnJlZSAlPiUgZmlsdGVyKFBwYXNfaW50cm9uID09IDAgJiByaWJvX2ludHJvbiA+PSAxNSkpICMgMjIxICAKCnBfaW50cm9uc19mcmVlX25vUEFfbm9SaWJvX2VucmljaCA8LSBwX2ludHJvbnNfZnJlZV9ub1BBX25vUmlibyAlPiUgZmlsdGVyKFBfb3Zlcl9DQl9pbnRyb25fbG9nID49IDApCnBfaW50cm9uc19mcmVlX25vUEFfbm9SaWJvX2RlcGxldCA8LSBwX2ludHJvbnNfZnJlZV9ub1BBX25vUmlibyAlPiUgZmlsdGVyKFBfb3Zlcl9DQl9pbnRyb25fbG9nIDwgMCkKCmNiX2ludHJvbnNfRUkgPC0gY2JfaW50cm9uc190aHJlc2ggJT4lIGZpbHRlcihtZWFuX2NiX2VpID49IDEgJiBtZWFuX2NiX2llIDwgMSkKY2JfaW50cm9uc19JRSA8LSBjYl9pbnRyb25zX3RocmVzaCAlPiUgZmlsdGVyKG1lYW5fY2JfaWUgPj0gMSAmIG1lYW5fY2JfZWkgPCAxKQpjYl9pbnRyb25zX2ZyZWUgPC0gY2JfaW50cm9uc190aHJlc2ggJT4lIGZpbHRlcihtZWFuX2NiX2llIDwgMSAmIG1lYW5fY2JfZWkgPCAxKQpjYl9pbnRyb25zX3JldGFpbmVkIDwtIGNiX2ludHJvbnNfdGhyZXNoICU+JSBmaWx0ZXIobWVhbl9jYl9pZSA+PSAxICYgbWVhbl9jYl9laSA+PSAxKQpjYl9pbnRyb25zX2ZyZWVfbm9QQV9ub1JpYm8gPC0gY2JfaW50cm9uc19mcmVlICU+JSBmaWx0ZXIoQ0JwYXNfaW50cm9uID09IDAgJiByaWJvX2ludHJvbiA8IDE1KQoKIyBDb21iaW5pbmcgaW50cm9uIHRhYmxlIHdpdGgga2FsbGlzdG8vc2xldXRoIG91dHB1dCAgCmludHJvbl9tYXN0ZXJfY2JwX2thbCA8LSBsZWZ0X2pvaW4oaW50cm9uX21hc3RlciwgY2JwNXJlcF9rYWwsIGJ5ID0gYygiZW5zX2dlbmVpZCIsImV4dF9nZW5laWQiKSkKcF9pbnRyb25zX2ZyZWVfbm9QQV9ub1JpYm8kaW50cm9udHlwZSA8LSBhcy5mYWN0b3IoImZyZWUiKQpwX2ludHJvbnNfcmV0YWluZWQkaW50cm9udHlwZSA8LSBhcy5mYWN0b3IoInJldGFpbmVkIikKcF9pbnRyb25zX2ZyZWVfcmV0YWluZWQgPC0gYmluZF9yb3dzKHBfaW50cm9uc19mcmVlX25vUEFfbm9SaWJvLCBwX2ludHJvbnNfcmV0YWluZWQpCnBfaW50cm9uc19mcmVlX3JldGFpbmVkX2NicF9rYWwgPC0gbGVmdF9qb2luKHBfaW50cm9uc19mcmVlX3JldGFpbmVkLCBjYnA1cmVwX2thbCwgYnkgPSBjKCJlbnNfZ2VuZWlkIiwiZXh0X2dlbmVpZCIpKQpgYGAKCgp+fn5+fn5+fn5+fn5+fn5+fn5+fn5+fn5+fiBQTE9UUyB+fn5+fn5+fn5+fn5+fn5+fn5+fn5+fn5+fn5+fn5+fn4KCmBgYHtyIGZpZy53aWR0aCA9IDgsIGZpZy5oZWlnaHQgPSA2LCByZXN1bHRzPSdoaWRlJywgZmlnLmtlZXA9J2FsbCcsIHdhcm5pbmcgPSBGQUxTRX0KZmlnMiA8LSBnZ3Bsb3QoY2JwNXJlcF9rYWwsIGFlcyhtZWFuX29icywgYikpICsKICBnZW9tX3BvaW50KGFscGhhID0gMC4wNSwgc2l6ZT0xLCBjb2xvcj0iZ3JheTMwIiwgc2hhcGUgPSAxNikgKyAKICBnZW9tX3ZsaW5lKHhpbnRlcmNlcHQ9MywgbGluZXR5cGU9ImRvdHRlZCIsIHNpemU9MC4xKSArIAogIGdlb21faGxpbmUoeWludGVyY2VwdD0wLCBsaW5ldHlwZT0iZG90dGVkIiwgc2l6ZT0wLjEpICsgCiAgZ2VvbV9wb2ludChkYXRhPWNicDVyZXBfa2FsW2NicDVyZXBfa2FsJHF2YWwgPCAwLjAxICYgY2JwNXJlcF9rYWwkYiA+MCxdLCBjb2xvcj0gIiNGRjY1MzIiLCBhbHBoYSA9IDAuNCwgc2l6ZSA9MSwgc2hhcGUgPSAxNikgKwogIGdlb21fcG9pbnQoZGF0YT1jYnA1cmVwX2thbFtjYnA1cmVwX2thbCRxdmFsIDwgMC4wMSAmIGNicDVyZXBfa2FsJGIgPDAsXSwgY29sb3I9ICIjMDA2NkNDIiwgYWxwaGEgPSAwLjQsIHNpemUgPTEsIHNoYXBlID0gMTYpICsKICBnZW9tX3BvaW50KGRhdGE9Y2JwNXJlcF9rYWxbY2JwNXJlcF9rYWwkbGFiZWxfcnBfbVJOQXMgPT0gVFJVRSAmIGNicDVyZXBfa2FsJHF2YWwgPCAwLjAxICYgY2JwNXJlcF9rYWwkbWVhbl9vYnMgPiAzLF0sIGNvbG9yPSAiYmxhY2siLCBhbHBoYSA9IDEsIHNpemUgPTEuMCkgKwogIGdlb21fcG9pbnQoZGF0YT1jYnA1cmVwX2thbFtjYnA1cmVwX2thbCRsYWJlbF9ycF9tUk5BcyA9PSBUUlVFICYgY2JwNXJlcF9rYWwkcXZhbCA8IDAuMDEgJiBjYnA1cmVwX2thbCRtZWFuX29icyA+IDMsXSwgY29sb3I9ICJibGFjayIsIGFscGhhID0gMSwgc2l6ZSA9MS4wKSArCiAgZ2VvbV9wb2ludChkYXRhPWNicDVyZXBfa2FsW2NicDVyZXBfa2FsJGxhYmVsX2RpZmZleHBfcm5hID09IFRSVUUsXSwgc2hhcGU9MjMsIHNpemUgPSAyLjUsIGNvbG9yPSAiYmxhY2siLCBzdHJva2U9MC40KSArCiAgZ2VvbV9sYWJlbF9yZXBlbChkYXRhPWNicDVyZXBfa2FsW2NicDVyZXBfa2FsJGxhYmVsX2RpZmZleHBfcm5hID09IFRSVUUsXSwgYWVzKGxhYmVsPWV4dF9nZW5laWQpLGNvbG9yPSAid2hpdGUiLCBzaXplPTMsIGZpbGwgPSAiZ3JheTQ3IiwgbWluLnNlZ21lbnQubGVuZ3RoID0gMCwgc2VnbWVudC5jb2xvdXIgPSAiYmxhY2siKSArCiAgeWxpbSgtNSw1KSArIHNjYWxlX3hfY29udGludW91cyhwb3NpdGlvbiA9ICJ0b3AiKSArCiAgeGxhYigiTWVhbiBsbihlc3QuIHJlYWQgY291bnRzKSIpICsgeWxhYigibG4ocHJvamVjdGlvbnMvd2hvbGUtY2VsbHMgZm9sZCBkaWZmZXJlbmNlKSIpICsKICB0aGVtZShwYW5lbC5ncmlkLm1ham9yID0gZWxlbWVudF9ibGFuaygpLCBwYW5lbC5ncmlkLm1pbm9yID0gZWxlbWVudF9ibGFuaygpLCBwYW5lbC5iYWNrZ3JvdW5kID0gZWxlbWVudF9ibGFuaygpLCBheGlzLmxpbmUgPSBlbGVtZW50X2xpbmUoY29sb3VyID0gJ2JsYWNrJywgc2l6ZSA9IDAuMikpCgpmaWcyCmBgYAoKYGBge3IgZmlnLndpZHRoID0gNiwgZmlnLmhlaWdodCA9IDUsIHJlc3VsdHM9J2hpZGUnLCBmaWcua2VlcD0nYWxsJ30KZmlnMyA8LSBnZ3Bsb3QoaW50cm9uX21hc3RlciwgYWVzKChtZWFuX2NiX2ludHJvbisxKSwgKG1lYW5fcF9pbnRyb24rMSkpKSArCiAgZ2VvbV9wb2ludChkYXRhPWludHJvbl9tYXN0ZXJbaW50cm9uX21hc3RlciRjYl90aHJlc2ggPT0gRiAmIGludHJvbl9tYXN0ZXIkcF90aHJlc2ggPT0gRixdLCBjb2xvcj0iZ3JheTMwIiwgYWxwaGEgPSAwLjEpICsgCiAgZ2VvbV9hYmxpbmUobGluZXR5cGU9ImRhc2hlZCIsIHNpemU9MC4yKSArIAogIGdlb21fcG9pbnQoZGF0YT1pbnRyb25fbWFzdGVyW2ludHJvbl9tYXN0ZXIkY2JfdGhyZXNoID09IFQsXSwgY29sb3I9IiMwMDY2Q0MiLCBhbHBoYSA9IDAuNSkgKwogIGdlb21fcG9pbnQoZGF0YT1pbnRyb25fbWFzdGVyW2ludHJvbl9tYXN0ZXIkcF90aHJlc2ggPT0gVCxdLCBjb2xvcj0iI0ZGNjUzMiIsIGFscGhhID0gMC41KSArCiAgZ2VvbV9wb2ludChkYXRhPWludHJvbl9tYXN0ZXJbaW50cm9uX21hc3RlciRpbnRyb25faWQgPT0gIkVOU1JOT0cwMDAwMDAxNjc3MF8wMDFpIixdLCBzaGFwZT0yMywgc2l6ZSA9IDMsIGNvbG9yPSAiYmxhY2siLCBzdHJva2U9MSkgKwogIGdlb21fcG9pbnQoZGF0YT1pbnRyb25fbWFzdGVyW2ludHJvbl9tYXN0ZXIkaW50cm9uX2lkID09ICJFTlNSTk9HMDAwMDAwMDU1MTNfMDA1aSIsXSwgc2hhcGU9MjMsIHNpemUgPSAzLCBjb2xvcj0gImJsYWNrIiwgc3Ryb2tlPTEpICsgICAgICAgCiAgZ2VvbV9sYWJlbF9yZXBlbChkYXRhPWludHJvbl9tYXN0ZXJbaW50cm9uX21hc3RlciRpbnRyb25faWQgPT0gIkVOU1JOT0cwMDAwMDAwNTUxM18wMDVpIiB8IGludHJvbl9tYXN0ZXIkaW50cm9uX2lkID09ICJFTlNSTk9HMDAwMDAwMTY3NzBfMDAxaSIsXSwgYWVzKGxhYmVsPWV4dF9nZW5laWQpLCBjb2xvcj0gIndoaXRlIiwgc2l6ZT0zLCBmaWxsID0gImdyYXk0NyIsIGFscGhhPTAuOSwgYm94LnBhZGRpbmcgPSAwLjI1LCBwb2ludC5wYWRkaW5nID0gMC4xKSArCiAgc2NhbGVfeF9sb2cxMChsaW1pdHM9YygxLDEwXjUpLCBicmVha3MgPSB0cmFuc19icmVha3MoImxvZzEwIiwgZnVuY3Rpb24oeCkgMTBeeCksIGxhYmVscyA9IHRyYW5zX2Zvcm1hdCgibG9nMTAiLCBtYXRoX2Zvcm1hdCgxMF4ueCkpKSArCiAgc2NhbGVfeV9sb2cxMChsaW1pdHM9YygxLDEwXjUpLCBicmVha3MgPSB0cmFuc19icmVha3MoImxvZzEwIiwgZnVuY3Rpb24oeCkgMTBeeCksIGxhYmVscyA9IHRyYW5zX2Zvcm1hdCgibG9nMTAiLCBtYXRoX2Zvcm1hdCgxMF4ueCkpKSArCiAgeGxhYigiTWVhbiByZWFkIGNvdW50cyAoV2hvbGUgY2VsbHMpIikgKyB5bGFiKCJNZWFuIHJlYWQgY291bnRzIChQcm9qZWN0aW9ucykiKSArCiAgdGhlbWUocGFuZWwuZ3JpZC5tYWpvciA9IGVsZW1lbnRfYmxhbmsoKSwgcGFuZWwuZ3JpZC5taW5vciA9IGVsZW1lbnRfYmxhbmsoKSwgcGFuZWwuYmFja2dyb3VuZCA9IGVsZW1lbnRfYmxhbmsoKSwgYXhpcy5saW5lID0gZWxlbWVudF9saW5lKGNvbG91ciA9ICJibGFjayIsIHNpemU9MC4yKSkgCgpmaWczCmBgYAoKCmBgYHtyIGZpZy53aWR0aCA9IDYsIGZpZy5oZWlnaHQgPSA1LCByZXN1bHRzPSdoaWRlJywgZmlnLmtlZXA9J2FsbCd9CmZpZzNfZmlnc3VwcDIgPC0gZ2dwbG90KGludHJvbl9tYXN0ZXIsIGFlcygobWVhbl9jYl9pbnRyb24rMSksIChtZWFuX3BfaW50cm9uKzEpKSkgKwogIGdlb21fcG9pbnQoZGF0YT1pbnRyb25fbWFzdGVyW2ludHJvbl9tYXN0ZXIkY2JfdGhyZXNoID09IEYgJiBpbnRyb25fbWFzdGVyJHBfdGhyZXNoID09IEYsXSwgY29sb3I9ImdyYXkzMCIsIGFscGhhID0gMC4xKSArIAogIGdlb21fYWJsaW5lKGxpbmV0eXBlPSJkYXNoZWQiLCBzaXplPTAuMikgKyAKICBnZW9tX3BvaW50KGRhdGE9aW50cm9uX21hc3RlcltpbnRyb25fbWFzdGVyJGNiX3RocmVzaCA9PSBULF0sIGNvbG9yPSIjMDA2NkNDIiwgYWxwaGEgPSAwLjUpICsKICBnZW9tX3BvaW50KGRhdGE9aW50cm9uX21hc3RlcltpbnRyb25fbWFzdGVyJHBfdGhyZXNoID09IFQsXSwgY29sb3I9IiNGRjY1MzIiLCBhbHBoYSA9IDAuNSkgKwogIGdlb21fcG9pbnQoZGF0YT1pbnRyb25fbWFzdGVyW2ludHJvbl9tYXN0ZXIkbGFiZWxfY2lydHMgPT0gVCAsXSwgY29sb3I9ImJsYWNrIikgKwogIGdlb21fbGFiZWxfcmVwZWwoZGF0YT1pbnRyb25fbWFzdGVyW2ludHJvbl9tYXN0ZXIkbGFiZWxfY2lydHMgPT0gVCAmIGludHJvbl9tYXN0ZXIkcF90aHJlc2ggPT0gVCwgXSwgYWVzKGxhYmVsPWV4dF9nZW5laWQpLCBjb2xvcj0gIndoaXRlIiwgc2l6ZT0zLCBmaWxsID0gImdyYXk0NyIsIG1pbi5zZWdtZW50Lmxlbmd0aCA9IDAsIHNlZ21lbnQuY29sb3VyID0gImJsYWNrIikgKwogIHNjYWxlX3hfbG9nMTAobGltaXRzPWMoMSwxMF41KSwgYnJlYWtzID0gdHJhbnNfYnJlYWtzKCJsb2cxMCIsIGZ1bmN0aW9uKHgpIDEwXngpLCBsYWJlbHMgPSB0cmFuc19mb3JtYXQoImxvZzEwIiwgbWF0aF9mb3JtYXQoMTBeLngpKSkgKwogIHNjYWxlX3lfbG9nMTAobGltaXRzPWMoMSwxMF41KSwgYnJlYWtzID0gdHJhbnNfYnJlYWtzKCJsb2cxMCIsIGZ1bmN0aW9uKHgpIDEwXngpLCBsYWJlbHMgPSB0cmFuc19mb3JtYXQoImxvZzEwIiwgbWF0aF9mb3JtYXQoMTBeLngpKSkgKwogIHhsYWIoIk1lYW4gcmVhZCBjb3VudHMgKFdob2xlIGNlbGxzKSIpICsgeWxhYigiTWVhbiByZWFkIGNvdW50cyAoUHJvamVjdGlvbnMpIikgKwogIHRoZW1lKHBhbmVsLmdyaWQubWFqb3IgPSBlbGVtZW50X2JsYW5rKCksIHBhbmVsLmdyaWQubWlub3IgPSBlbGVtZW50X2JsYW5rKCksIHBhbmVsLmJhY2tncm91bmQgPSBlbGVtZW50X2JsYW5rKCksIGF4aXMubGluZSA9IGVsZW1lbnRfbGluZShjb2xvdXIgPSAiYmxhY2siLCBzaXplPTAuMikpIAoKZmlnM19maWdzdXBwMgpgYGAKCgpgYGB7ciBmaWcud2lkdGggPSA3LCBmaWcuaGVpZ2h0ID0gNiwgcmVzdWx0cz0naGlkZScsIGZpZy5rZWVwPSdhbGwnfQpmaWc0IDwtIGdncGxvdChwX2ludHJvbnNfdGhyZXNoLCBhZXMobWVhbl9wX2llKzAuMSwgbWVhbl9wX2VpKzAuMSkpICsKICBnZW9tX3BvaW50KGFscGhhID0gMC43LCBjb2xvcj0iZ3JheTMwIikgKyAKICBnZW9tX2hsaW5lKHlpbnRlcmNlcHQ9MSwgbGluZXR5cGU9ImRhc2hlZCIsIHNpemUgPSAwLjUpICsgCiAgZ2VvbV92bGluZSh4aW50ZXJjZXB0PTEsIGxpbmV0eXBlPSJkYXNoZWQiLCBzaXplPTAuNSkgKyAKICBnZW9tX3BvaW50KGRhdGE9cF9pbnRyb25zX3RocmVzaFtwX2ludHJvbnNfdGhyZXNoJGludHJvbl9pZCAgPT0gIkVOU1JOT0cwMDAwMDAxNjc3MF8wMDFpIixdLCBzaGFwZT0yMywgc2l6ZSA9IDMsIGNvbG9yPSAiYmxhY2siLCBzdHJva2U9MSkgKyAjQ2FsbTIKICBnZW9tX3BvaW50KGRhdGE9cF9pbnRyb25zX3RocmVzaFtwX2ludHJvbnNfdGhyZXNoJGludHJvbl9pZCAgPT0gIkVOU1JOT0cwMDAwMDAwNzY4Nl8wMTBpIixdLCBzaGFwZT0yMywgc2l6ZSA9IDMsIGNvbG9yPSAiYmxhY2siLCBzdHJva2U9MSkgKyAjIFNlcHQzCiAgZ2VvbV9wb2ludChkYXRhPXBfaW50cm9uc190aHJlc2hbcF9pbnRyb25zX3RocmVzaCRpbnRyb25faWQgID09ICJFTlNSTk9HMDAwMDAwMDYyMzVfMDIyaSIsXSwgc2hhcGU9MjMsIHNpemUgPSAzLCBjb2xvcj0gImJsYWNrIiwgc3Ryb2tlPTEpICsgIyBOZWxsMgogIGdlb21fcG9pbnQoZGF0YT1wX2ludHJvbnNfdGhyZXNoW3BfaW50cm9uc190aHJlc2gkaW50cm9uX2lkICA9PSAiRU5TUk5PRzAwMDAwMDE3NTAwXzAwNWkiLF0sIHNoYXBlPTIzLCBzaXplID0gMywgY29sb3I9ICJibGFjayIsIHN0cm9rZT0xKSArICMgTXRzczFsCiAgZ2VvbV9wb2ludChkYXRhPXBfaW50cm9uc190aHJlc2hbcF9pbnRyb25zX3RocmVzaCRpbnRyb25faWQgID09ICJFTlNSTk9HMDAwMDAwMDE1NTdfMDA3aSIsXSwgc2hhcGU9MjMsIHNpemUgPSAzLCBjb2xvcj0gImJsYWNrIiwgc3Ryb2tlPTEpICsKICBnZW9tX3BvaW50KGRhdGE9cF9pbnRyb25zX3RocmVzaFtwX2ludHJvbnNfdGhyZXNoJGludHJvbl9pZCAgPT0gIkVOU1JOT0cwMDAwMDAyMDg1MV8wMDVpIixdLCBzaGFwZT0yMywgc2l6ZSA9IDMsIGNvbG9yPSAiYmxhY2siLCBzdHJva2U9MSkgKwogIGdlb21fcG9pbnQoZGF0YT1wX2ludHJvbnNfdGhyZXNoW3BfaW50cm9uc190aHJlc2gkaW50cm9uX2lkICA9PSAiRU5TUk5PRzAwMDAwMDU1MzAwXzAyOWkiLF0sIHNoYXBlPTIzLCBzaXplID0gMywgY29sb3I9ICJibGFjayIsIHN0cm9rZT0xKSArICMgTmNhcGQyICAKICBnZW9tX2xhYmVsX3JlcGVsKGRhdGE9cF9pbnRyb25zX3RocmVzaFtwX2ludHJvbnNfdGhyZXNoJGludHJvbl9pZCA9PSAiRU5TUk5PRzAwMDAwMDE2NzcwXzAwMWkiLCBdLCBhZXMobGFiZWw9ZXh0X2dlbmVpZCksICBjb2xvcj0gIndoaXRlIiwgc2l6ZT0zLCBmaWxsID0gImdyYXk0NyIsIG1pbi5zZWdtZW50Lmxlbmd0aCA9IDAsIHNlZ21lbnQuY29sb3VyID0gImJsYWNrIikgKwogIGdlb21fbGFiZWxfcmVwZWwoZGF0YT1wX2ludHJvbnNfdGhyZXNoW3BfaW50cm9uc190aHJlc2gkaW50cm9uX2lkID09ICJFTlNSTk9HMDAwMDAwMDc2ODZfMDEwaSIsIF0sIGFlcyhsYWJlbD1leHRfZ2VuZWlkKSwgIGNvbG9yPSAid2hpdGUiLCBzaXplPTMsIGZpbGwgPSAiZ3JheTQ3IiwgbWluLnNlZ21lbnQubGVuZ3RoID0gMCwgc2VnbWVudC5jb2xvdXIgPSAiYmxhY2siKSArCiAgZ2VvbV9sYWJlbF9yZXBlbChkYXRhPXBfaW50cm9uc190aHJlc2hbcF9pbnRyb25zX3RocmVzaCRpbnRyb25faWQgPT0gIkVOU1JOT0cwMDAwMDAwNjIzNV8wMjJpIiwgXSwgYWVzKGxhYmVsPWV4dF9nZW5laWQpLCAgY29sb3I9ICJ3aGl0ZSIsIHNpemU9MywgZmlsbCA9ICJncmF5NDciLCBtaW4uc2VnbWVudC5sZW5ndGggPSAwLCBzZWdtZW50LmNvbG91ciA9ICJibGFjayIpICsKICBnZW9tX2xhYmVsX3JlcGVsKGRhdGE9cF9pbnRyb25zX3RocmVzaFtwX2ludHJvbnNfdGhyZXNoJGludHJvbl9pZCA9PSAiRU5TUk5PRzAwMDAwMDE3NTAwXzAwNWkiLCBdLCBhZXMobGFiZWw9ZXh0X2dlbmVpZCksICBjb2xvcj0gIndoaXRlIiwgc2l6ZT0zLCBmaWxsID0gImdyYXk0NyIsIG1pbi5zZWdtZW50Lmxlbmd0aCA9IDAsIHNlZ21lbnQuY29sb3VyID0gImJsYWNrIikgKwogIGdlb21fbGFiZWxfcmVwZWwoZGF0YT1wX2ludHJvbnNfdGhyZXNoW3BfaW50cm9uc190aHJlc2gkaW50cm9uX2lkID09ICJFTlNSTk9HMDAwMDAwMDE1NTdfMDA3aSIsIF0sIGFlcyhsYWJlbD1leHRfZ2VuZWlkKSwgIGNvbG9yPSAid2hpdGUiLCBzaXplPTMsIGZpbGwgPSAiZ3JheTQ3IiwgbWluLnNlZ21lbnQubGVuZ3RoID0gMCwgc2VnbWVudC5jb2xvdXIgPSAiYmxhY2siKSArCiAgZ2VvbV9sYWJlbF9yZXBlbChkYXRhPXBfaW50cm9uc190aHJlc2hbcF9pbnRyb25zX3RocmVzaCRpbnRyb25faWQgPT0gIkVOU1JOT0cwMDAwMDAyMDg1MV8wMDVpIiwgXSwgYWVzKGxhYmVsPWV4dF9nZW5laWQpLCAgY29sb3I9ICJ3aGl0ZSIsIHNpemU9MywgZmlsbCA9ICJncmF5NDciLCBtaW4uc2VnbWVudC5sZW5ndGggPSAwLCBzZWdtZW50LmNvbG91ciA9ICJibGFjayIpICsKICBnZW9tX2xhYmVsX3JlcGVsKGRhdGE9cF9pbnRyb25zX3RocmVzaFtwX2ludHJvbnNfdGhyZXNoJGludHJvbl9pZCA9PSAiRU5TUk5PRzAwMDAwMDU1MzAwXzAyOWkiLCBdLCBhZXMobGFiZWw9ZXh0X2dlbmVpZCksICBjb2xvcj0gIndoaXRlIiwgc2l6ZT0zLCBmaWxsID0gImdyYXk0NyIsIG1pbi5zZWdtZW50Lmxlbmd0aCA9IDAsIHNlZ21lbnQuY29sb3VyID0gImJsYWNrIikgKwogIHNjYWxlX3hfbG9nMTAobGltaXRzPWMoMC4xLDMwMDAwKSkgKyBzY2FsZV95X2xvZzEwKGxpbWl0cz1jKDAuMSwzMDAwMCkpICsKICB4bGFiKCJNZWFuIGludHJvbi1leG9uIHJlYWRzIikgKyB5bGFiKCJNZWFuIGV4b24taW50cm9uIHJlYWRzIikgKwogIHRoZW1lKHBhbmVsLmdyaWQubWFqb3IgPSBlbGVtZW50X2JsYW5rKCksIHBhbmVsLmdyaWQubWlub3IgPSBlbGVtZW50X2JsYW5rKCksIHBhbmVsLmJhY2tncm91bmQgPSBlbGVtZW50X2JsYW5rKCksIGF4aXMubGluZSA9IGVsZW1lbnRfbGluZShjb2xvdXIgPSAiYmxhY2siLCBzaXplPTAuMikpCgpmaWc0CmBgYAoKYGBge3IgZmlnLndpZHRoID0gOCwgZmlnLmhlaWdodCA9IDcsIHJlc3VsdHM9J2hpZGUnLCBmaWcua2VlcD0nYWxsJywgd2FybmluZyA9IEZBTFNFfQpmaWc1IDwtIGdncGxvdChwX2ludHJvbnNfZnJlZV9ub1BBX25vUmlibywgYWVzKChjYl9pbnRyb25fcGVybGVuK3BfaW50cm9uX3BlcmxlbikvMiwgUF9vdmVyX0NCX2ludHJvbl9sb2cpKSArCiAgZ2VvbV9wb2ludChhbHBoYSA9IDAuNywgY29sb3I9ImdyYXkzMCIsIHNpemUgPSAxKSArIGdlb21faGxpbmUoeWludGVyY2VwdD0gMCwgbGluZXR5cGU9ImRhc2hlZCIsIHNpemU9MC4yKSArCiAgZ2VvbV9wb2ludChkYXRhID0gcF9pbnRyb25zX2ZyZWVfbm9QQV9ub1JpYm9bcF9pbnRyb25zX2ZyZWVfbm9QQV9ub1JpYm8kY2lyY19udW1fcCA+PSAyICYgcF9pbnRyb25zX2ZyZWVfbm9QQV9ub1JpYm8kUF9vdmVyX0NCX2ludHJvbl9sb2cgPiAwLF0sIGFscGhhID0gMSwgY29sb3I9InJlZCIsIHNpemUgPSAxKSArCiAgZ2VvbV9wb2ludChkYXRhID0gcF9pbnRyb25zX2ZyZWVfbm9QQV9ub1JpYm9bcF9pbnRyb25zX2ZyZWVfbm9QQV9ub1JpYm8kaW50cm9uX2lkID09ICJFTlNSTk9HMDAwMDAwMDMyMDdfMDI3aSIgfCBwX2ludHJvbnNfZnJlZV9ub1BBX25vUmlibyRpbnRyb25faWQgPT0gIkVOU1JOT0cwMDAwMDA1NzA3OF8wMDFpIiB8IHBfaW50cm9uc19mcmVlX25vUEFfbm9SaWJvJGludHJvbl9pZCA9PSAiRU5TUk5PRzAwMDAwMDA3NjIyXzAwNmkiLF0sIGFscGhhID0gMSwgY29sb3I9InJlZCIsIHNpemUgPSAxKSArCiAgZ2VvbV9sYWJlbF9yZXBlbChkYXRhID0gcF9pbnRyb25zX2ZyZWVfbm9QQV9ub1JpYm9bcF9pbnRyb25zX2ZyZWVfbm9QQV9ub1JpYm8kY2lyY19udW1fcCA+PSAyICYgcF9pbnRyb25zX2ZyZWVfbm9QQV9ub1JpYm8kUF9vdmVyX0NCX2ludHJvbl9sb2cgPiAwLF0sIGFlcyhsYWJlbD1leHRfZ2VuZWlkKSwgY29sb3I9ICJ3aGl0ZSIsIHNpemU9MywgZmlsbCA9ICJncmF5NDciLCBtaW4uc2VnbWVudC5sZW5ndGggPSAwLCBzZWdtZW50LmNvbG91ciA9ICJibGFjayIpICsKICBnZW9tX2xhYmVsX3JlcGVsKGRhdGEgPSBwX2ludHJvbnNfZnJlZV9ub1BBX25vUmlib1twX2ludHJvbnNfZnJlZV9ub1BBX25vUmlibyRpbnRyb25faWQgPT0gIkVOU1JOT0cwMDAwMDAwMzIwN18wMjdpIiB8IHBfaW50cm9uc19mcmVlX25vUEFfbm9SaWJvJGludHJvbl9pZCA9PSAiRU5TUk5PRzAwMDAwMDU3MDc4XzAwMWkiIHwgcF9pbnRyb25zX2ZyZWVfbm9QQV9ub1JpYm8kaW50cm9uX2lkID09ICJFTlNSTk9HMDAwMDAwMDc2MjJfMDA2aSIsXSwgYWVzKGxhYmVsPWV4dF9nZW5laWQpLCBjb2xvcj0gIndoaXRlIiwgc2l6ZT0zLCBmaWxsID0gImdyYXk0NyIsIG1pbi5zZWdtZW50Lmxlbmd0aCA9IDAsIHNlZ21lbnQuY29sb3VyID0gImJsYWNrIikgKwogIGdlb21fbGFiZWxfcmVwZWwoZGF0YSA9IHBfaW50cm9uc19mcmVlX25vUEFfbm9SaWJvW3BfaW50cm9uc19mcmVlX25vUEFfbm9SaWJvJGludHJvbl9pZCA9PSAiRU5TUk5PRzAwMDAwMDA1NTEzXzAwNWkiIHwgcF9pbnRyb25zX2ZyZWVfbm9QQV9ub1JpYm8kaW50cm9uX2lkID09ICJFTlNSTk9HMDAwMDAwMjU3NjhfMDEwaSIgfCBwX2ludHJvbnNfZnJlZV9ub1BBX25vUmlibyRpbnRyb25faWQgPT0gIkVOU1JOT0cwMDAwMDA1NTMwMF8wMjlpIiB8IHBfaW50cm9uc19mcmVlX25vUEFfbm9SaWJvJGludHJvbl9pZCA9PSAiRU5TUk5PRzAwMDAwMDEwNzY4XzAwNWkiIHwgcF9pbnRyb25zX2ZyZWVfbm9QQV9ub1JpYm8kaW50cm9uX2lkID09ICJFTlNSTk9HMDAwMDAwMDk3MjJfMDEyaSIgfCBwX2ludHJvbnNfZnJlZV9ub1BBX25vUmlibyRpbnRyb25faWQgPT0gIkVOU1JOT0cwMDAwMDAxMjk5OV8wMDdpIixdLCBhZXMobGFiZWw9ZXh0X2dlbmVpZCksIGNvbG9yPSAid2hpdGUiLCBzaXplPTMsIGZpbGwgPSAiZ3JheTQ3IiwgbWluLnNlZ21lbnQubGVuZ3RoID0gMCwgc2VnbWVudC5jb2xvdXIgPSAiYmxhY2siKSArCiAgc2NhbGVfeF9sb2cxMCgpICsgeWxpbSgtNSw1KSArCiAgeGxhYigiTWVhbiByZWFkIGRlbnNpdHkgcGVyIGludHJvbiIpICsgeWxhYigibG4gKHByb2plY3Rpb25zL3dob2xlLWNlbGxzKSByZWFkIGNvdW50cyIpICsKICB0aGVtZShwYW5lbC5ncmlkLm1ham9yID0gZWxlbWVudF9ibGFuaygpLCBwYW5lbC5ncmlkLm1pbm9yID0gZWxlbWVudF9ibGFuaygpLCBwYW5lbC5iYWNrZ3JvdW5kID0gZWxlbWVudF9ibGFuaygpLCBheGlzLmxpbmUgPSBlbGVtZW50X2xpbmUoY29sb3VyID0gImJsYWNrIiwgc2l6ZT0wLjIpKQoKZmlnNQpgYGAKCmBgYHtyIGZpZy53aWR0aCA9IDYsIGZpZy5oZWlnaHQgPSA0LCByZXN1bHRzPSdoaWRlJywgZmlnLmtlZXA9J2FsbCcsIHdhcm5pbmcgPSBGQUxTRX0KZmlnNmMgPC0gZ2dwbG90KHBfcmV0YWluZWRfZW5yaWNoX2RlcGxldF9leG9uc19waHlsb1AsIGFlcyh4PW1lZGlhbl9jb25zLCBjb2xvcj1zYW1wbGUpKSArIHN0YXRfZWNkZigpICsKICB4bGltKC0wLjUsNCkgKwogIHhsYWIoIk1lZGlhbiBwaHlsb1Agc2NvcmUiKSArIHlsYWIoIkZyYWN0aW9uIG9mIGRhdGEiKSArCiAgdGhlbWUocGFuZWwuZ3JpZC5tYWpvciA9IGVsZW1lbnRfYmxhbmsoKSwgcGFuZWwuZ3JpZC5taW5vciA9IGVsZW1lbnRfYmxhbmsoKSwgcGFuZWwuYmFja2dyb3VuZCA9IGVsZW1lbnRfYmxhbmsoKSwgYXhpcy5saW5lID0gZWxlbWVudF9saW5lKGNvbG91ciA9ICJibGFjayIsIHNpemU9MC4yKSkgCgpmaWc2YwpgYGAKCgpgYGB7ciBmaWcud2lkdGggPSA2LCBmaWcuaGVpZ2h0ID0gNSwgcmVzdWx0cz0naGlkZScsIGZpZy5rZWVwPSdhbGwnLCB3YXJuaW5nID0gRkFMU0V9CmZpZzRfZmlnc3VwcDJBIDwtIGdncGxvdChwX2ludHJvbnNfZnJlZV9yZXRhaW5lZF9jYnBfa2FsW3BfaW50cm9uc19mcmVlX3JldGFpbmVkX2NicF9rYWwkaW50cm9udHlwZSA9PSAiZnJlZSIsXSwgYWVzKGIsIFBfb3Zlcl9DQl9pbnRyb25fbG9nKSkgKwogIGdlb21fcG9pbnQoYWxwaGEgPSAwLjUsIHNpemU9MC41LCBjb2xvcj0iZ3JheTMwIikgKyAKICBnZW9tX2hsaW5lKHlpbnRlcmNlcHQ9MCwgbGluZXR5cGU9ImRhc2hlZCIsIHNpemU9MC4yKSArIGdlb21fdmxpbmUoeGludGVyY2VwdD0gMCwgbGluZXR5cGU9ImRhc2hlZCIsIHNpemU9MC4yKSArCiAgZ2VvbV9wb2ludChkYXRhID0gcF9pbnRyb25zX2ZyZWVfcmV0YWluZWRfY2JwX2thbFtwX2ludHJvbnNfZnJlZV9yZXRhaW5lZF9jYnBfa2FsJGludHJvbnR5cGUgPT0gImZyZWUiICYgcF9pbnRyb25zX2ZyZWVfcmV0YWluZWRfY2JwX2thbCRjaXJjX251bV9wID49IDIgJiBwX2ludHJvbnNfZnJlZV9yZXRhaW5lZF9jYnBfa2FsJFBfb3Zlcl9DQl9pbnRyb25fbG9nID4gMCxdLCBhbHBoYSA9IDEsIGNvbG9yPSJyZWQiLCBzaXplID0gMSkgKwogIGdlb21fcG9pbnQoZGF0YSA9IHBfaW50cm9uc19mcmVlX3JldGFpbmVkX2NicF9rYWxbcF9pbnRyb25zX2ZyZWVfcmV0YWluZWRfY2JwX2thbCRpbnRyb250eXBlID09ICJmcmVlIiAmIHBfaW50cm9uc19mcmVlX3JldGFpbmVkX2NicF9rYWwkaW50cm9uX2lkID09ICJFTlNSTk9HMDAwMDAwMDMyMDdfMDI3aSIgfCBwX2ludHJvbnNfZnJlZV9yZXRhaW5lZF9jYnBfa2FsJGludHJvbl9pZCA9PSAiRU5TUk5PRzAwMDAwMDU3MDc4XzAwMWkiIHwgcF9pbnRyb25zX2ZyZWVfcmV0YWluZWRfY2JwX2thbCRpbnRyb25faWQgPT0gIkVOU1JOT0cwMDAwMDAwNzYyMl8wMDZpIixdLCBhbHBoYSA9IDEsIGNvbG9yPSJyZWQiLCBzaXplID0gMSkgKwogIHhsaW0oLTUsNSkgKyB5bGltKC01LDUpICsKICB4bGFiKCJsbiAocHJvamVjdGlvbnMvd2hvbGUtY2VsbHMpIG1STkEgZm9sZCBkaWZmZXJlbmNlIikgKyB5bGFiKCJsbiAocHJvamVjdGlvbnMvd2hvbGUtY2VsbHMpIHJlYWRzIHBlciBpbnRyb24gcmVnaW9uIikgKwogIGdndGl0bGUoIkZyZWUgaW50cm9uIHZlcnN1cyBtUk5BIGVucmljaG1lbnQgaW4gcHJvamVjdGlvbnMiKSArCiAgdGhlbWUocGFuZWwuZ3JpZC5tYWpvciA9IGVsZW1lbnRfYmxhbmsoKSwgcGFuZWwuZ3JpZC5taW5vciA9IGVsZW1lbnRfYmxhbmsoKSwgcGFuZWwuYmFja2dyb3VuZCA9IGVsZW1lbnRfYmxhbmsoKSwgYXhpcy5saW5lID0gZWxlbWVudF9saW5lKGNvbG91ciA9ICJibGFjayIsIHNpemU9MC4yKSkKCmZpZzRfZmlnc3VwcDJBCmBgYAoKYGBge3IgZmlnLndpZHRoID0gNiwgZmlnLmhlaWdodCA9IDUsIHJlc3VsdHM9J2hpZGUnLCBmaWcua2VlcD0nYWxsJywgd2FybmluZyA9IEZBTFNFfQpmaWc0X2ZpZ3N1cHAyQiA8LSBnZ3Bsb3QocF9pbnRyb25zX2ZyZWVfcmV0YWluZWRfY2JwX2thbFtwX2ludHJvbnNfZnJlZV9yZXRhaW5lZF9jYnBfa2FsJGludHJvbnR5cGUgPT0gInJldGFpbmVkIixdLCBhZXMoYiwgUF9vdmVyX0NCX2ludHJvbl9sb2cpKSArCiAgZ2VvbV9wb2ludChhbHBoYSA9IDAuNSwgc2l6ZT0wLjUsIGNvbG9yPSJncmF5MzAiKSArIAogIGdlb21faGxpbmUoeWludGVyY2VwdD0wLCBsaW5ldHlwZT0iZGFzaGVkIiwgc2l6ZT0wLjIpICsgZ2VvbV92bGluZSh4aW50ZXJjZXB0PSAwLCBsaW5ldHlwZT0iZGFzaGVkIiwgc2l6ZT0wLjIpICsKICBnZW9tX3BvaW50KGRhdGE9cF9pbnRyb25zX2ZyZWVfcmV0YWluZWRfY2JwX2thbFtwX2ludHJvbnNfZnJlZV9yZXRhaW5lZF9jYnBfa2FsJGludHJvbnR5cGUgPT0gInJldGFpbmVkIiAmIHBfaW50cm9uc19mcmVlX3JldGFpbmVkX2NicF9rYWwkZXh0X2dlbmVpZCA9PSAiU2VwdDMiLF0sIHNpemUgPSAxLCBjb2xvcj0gImJsYWNrIikgKwogIGdlb21fcG9pbnQoZGF0YT1wX2ludHJvbnNfZnJlZV9yZXRhaW5lZF9jYnBfa2FsW3BfaW50cm9uc19mcmVlX3JldGFpbmVkX2NicF9rYWwkaW50cm9udHlwZSA9PSAicmV0YWluZWQiICYgcF9pbnRyb25zX2ZyZWVfcmV0YWluZWRfY2JwX2thbCRpbnRyb25faWQgPT0gIkVOU1JOT0cwMDAwMDAxNjc3MF8wMDFpIixdLCBzaXplID0gMSwgY29sb3I9ICJibGFjayIpICsKICBnZW9tX2xhYmVsX3JlcGVsKGRhdGEgPSBwX2ludHJvbnNfZnJlZV9yZXRhaW5lZF9jYnBfa2FsW3BfaW50cm9uc19mcmVlX3JldGFpbmVkX2NicF9rYWwkaW50cm9udHlwZSA9PSAicmV0YWluZWQiICYgcF9pbnRyb25zX2ZyZWVfcmV0YWluZWRfY2JwX2thbCRpbnRyb25faWQgPT0gIkVOU1JOT0cwMDAwMDAxNjc3MF8wMDFpIiB8IHBfaW50cm9uc19mcmVlX3JldGFpbmVkX2NicF9rYWwkZXh0X2dlbmVpZCA9PSAiU2VwdDMiLF0sIGFlcyhsYWJlbD1leHRfZ2VuZWlkKSwgY29sb3I9ICJ3aGl0ZSIsIHNpemU9MywgZmlsbCA9ICJncmF5NDciKSArCiAgeGxpbSgtNSw1KSArIHlsaW0oLTUsNSkgKwogIHhsYWIoImxuIChwcm9qZWN0aW9ucy93aG9sZS1jZWxscykgbVJOQSBmb2xkIGRpZmZlcmVuY2UiKSArIHlsYWIoImxuIChwcm9qZWN0aW9ucy93aG9sZS1jZWxscykgcmVhZHMgcGVyIGludHJvbiByZWdpb24iKSArCiAgZ2d0aXRsZSgiUmV0YWluZWQgaW50cm9uIHZlcnN1cyBtUk5BIGVucmljaG1lbnQgaW4gcHJvamVjdGlvbnMiKSArCiAgdGhlbWUocGFuZWwuZ3JpZC5tYWpvciA9IGVsZW1lbnRfYmxhbmsoKSwgcGFuZWwuZ3JpZC5taW5vciA9IGVsZW1lbnRfYmxhbmsoKSwgcGFuZWwuYmFja2dyb3VuZCA9IGVsZW1lbnRfYmxhbmsoKSwgYXhpcy5saW5lID0gZWxlbWVudF9saW5lKGNvbG91ciA9ICJibGFjayIsIHNpemU9MC4yKSkKCmZpZzRfZmlnc3VwcDJCCgojbnJvdyhwX2ludHJvbnNfZnJlZV9yZXRhaW5lZF9jYnBfa2FsICU+JSBmaWx0ZXIoaW50cm9udHlwZSA9PSAicmV0YWluZWQiKSAlPiUgIAojICAgICAgIGZpbHRlcihQX292ZXJfQ0JfaW50cm9uX2xvZyA+IDAgJiBiIDw9IDApKSAgCiNucm93KHBfaW50cm9uc19mcmVlX3JldGFpbmVkX2NicF9rYWwgJT4lIGZpbHRlcihpbnRyb250eXBlID09ICJmcmVlIikgJT4lICAKIyAgICAgICBmaWx0ZXIoUF9vdmVyX0NCX2ludHJvbl9sb2cgPD0gMCAmIGIgPiAwKSkgIAojIHVwcGVyIHJpZ2h0ID0gMTIwIChyZXRhaW5lZCkgYW5kIDMxIChmcmVlKSAgCiMgdXBwZXIgbGVmdCA9IDYgYW5kIDIyICAKIyBsb3dlciByaWdodCA9IDI4MCBhbmQgMTQ2IAojIGxvd2VyIGxlZnQgPSAyMzIgYW5kIDE4MyAgCmBgYAoKCmBgYHtyIGZpZy53aWR0aCA9IDUsIGZpZy5oZWlnaHQgPSA1LCBlY2hvPUZBTFNFLCByZXN1bHRzPSdoaWRlJywgZmlnLmtlZXA9J2FsbCcsIHdhcm5pbmcgPSBGQUxTRX0Kcm5hc2VxX3NhbXBsZXMgPC0gc2FtcGxlc1sgc2FtcGxlcyRMaWJyYXJ5LnR5cGUgPT0gIlJOQS1TZXEiLF0KCnNhbXBsZV9uYW1lcyA8LSB0KGFzLm1hdHJpeChybmFzZXFfc2FtcGxlcyRTYW1wbGUuLmNvdW50cy5wZXIubWlsbGlvbi4pKQpzYW1wbGVfbmFtZXMKCmxlbmd0aChzYW1wbGVfbmFtZXMpCgojIENyZWF0ZSBhbiBlbXB0eSBtYXRyaXggdG8gZmlsbCB3aXRoIFNwZWFybWFuIGNvZWZmaWNpZW50cyAgCmNvcnJfbWF0X2NicDVyZXAgPC0gbWF0cml4KGRhdGEgPSBOQSwgbnJvdz1sZW5ndGgoc2FtcGxlX25hbWVzKSwgbmNvbD1sZW5ndGgoc2FtcGxlX25hbWVzKSkKY29sbmFtZXMoY29ycl9tYXRfY2JwNXJlcCkgPC0gYygicHJval9yMSIsICJwcm9qX3IyIiwgInByb2pfcjMiLCAicHJval9yNCIsICJwcm9qX3I1IiwgIndoY2VsbF9yMSIsICJ3aGNlbGxfcjIiLCAid2hjZWxsX3IzIiwgIndoY2VsbF9yNCIsICJ3aGNlbGxfcjUiKQpyb3duYW1lcyhjb3JyX21hdF9jYnA1cmVwKSA8LSBjKCJwcm9qX3IxIiwgInByb2pfcjIiLCAicHJval9yMyIsICJwcm9qX3I0IiwgInByb2pfcjUiLCAid2hjZWxsX3IxIiwgIndoY2VsbF9yMiIsICJ3aGNlbGxfcjMiLCAid2hjZWxsX3I0IiwgIndoY2VsbF9yNSIpCmRpbShjb3JyX21hdF9jYnA1cmVwKQpoZWFkKGNvcnJfbWF0X2NicDVyZXApCgpmb3IgKCBpIGluIDE6bGVuZ3RoKHNhbXBsZV9uYW1lcykpIAogICAgICAgICAgICB7IGZvciAoaiBpbiAxOmxlbmd0aChzYW1wbGVfbmFtZXMpKSAKICAgICAgICAgICAgICB7IGNvcnIgPC0gY29yLnRlc3QoY2JwNXJlcF9rYWxbLHNhbXBsZV9uYW1lc1tpXV0sIGNicDVyZXBfa2FsWyxzYW1wbGVfbmFtZXNbal1dLCBtZXRob2Q9InNwZWFybWFuIiwgbmEucm09VCkKICAgICAgICAgICAgICAgIGNvcnJfbWF0X2NicDVyZXBbaSxqXSA8LSByb3VuZChjb3JyJGVzdGltYXRlLCAyKQogICAgICAgICAgICAgICAgICAgICAgfQogICAgICAgICAgICAgIH0KCmNvcnJfbWF0X2NicDVyZXAKCmBgYApgYGB7ciBmaWcud2lkdGggPSA1LCBmaWcuaGVpZ2h0ID0gNSwgcmVzdWx0cz0naGlkZScsIGZpZy5rZWVwPSdhbGwnfQpmaWcyX2ZpZ3N1cHAxIDwtIHBoZWF0bWFwKGNvcnJfbWF0X2NicDVyZXAsIHNjYWxlID0gIm5vbmUiLAogICAgICAgICBmb250c2l6ZV9yb3cgPSA4LCAKICAgICAgICAgZm9udHNpemVfY29sID0gOCwKICAgICAgICAgY2VsbHdpZHRoPTIwLAogICAgICAgICBjZWxsaGVpZ2h0ID0gMjAsCiAgICAgICAgIGNvbG9yID0gY29sb3JSYW1wUGFsZXR0ZShicmV3ZXIucGFsKG49OCwgbmFtZSA9ICJZbE9yUmQiKSkoNTApLAogICAgICAgICBjbHVzdGVyX2NvbHM9RiwKICAgICAgICAgY2x1c3Rlcl9yb3dzPUYsCiAgICAgICAgIGNsdXN0ZXJpbmdfZGlzdGFuY2VfY29scyA9ICJldWNsaWRlYW4iLCAKICAgICAgICAgY2x1c3RlcmluZ19tZXRob2QgPSAiY29tcGxldGUiLAogICAgICAgICBhbm5vdGF0aW9uX2xlZ2VuZCA9IFRSVUUsIAogICAgICAgICBkaXNwbGF5X251bWJlcnM9IFQpCgpmaWcyX2ZpZ3N1cHAxCmBgYAoKYGBge3J9CnNlc3Npb25JbmZvKCkKYGBgCg==
