# Supplementary material for: Free circular introns with an unusual branchpoint in neuronal projections
Source: eLife. 2019 Nov 7;8:e47809. doi: 10.7554/eLife.47809 (PMC6879206; doi:10.7554/eLife.47809)
Supplement: Source code 1. [file elife-47809-code1.gz › Saini19-supplement/code_for_saini_et_al_19/Rscript_sleuth_RNA_diffexp.nb.html]

RNA differential expression analysis for whole-cells v/s projections


Code 

- Show All Code
- Hide All Code
- Download Rmd

# RNA differential expression analysis for whole-cells v/s projections

#### *Harleen Saini*

#### *October 19, 2019*


```
library(shiny)
library(sleuth)
library(reshape2)
library(dplyr)
```


```
# setting the path to Kallisto results in "base_dir"  
base_dir <- "../data_tables_for_saini_et_al_19/Intermediate_tables_to_reproduce_figures/kallisto_output_for_diff_expression_by_sleuth/"
# To include gene names into transcript-level analysis, pulling Ensembl data using biomaRt:  
mart <- biomaRt::useMart(biomart = "ENSEMBL_MART_ENSEMBL",
                         dataset = "rnorvegicus_gene_ensembl",
                         host = "useast.ensembl.org")
t2g_ens <- biomaRt::getBM(attributes = c("ensembl_transcript_id", "ensembl_gene_id",
                                         "external_gene_name"), mart = mart)
# Xist is not annotated in the rat rnor6 genome.  
# Used LiftOver (UCSC/Kent Tools) to get rat Xist sequence for input to Kallisto  
# Assigned artifical ensembl IDs for the gene and transcript to enter into Sleuth  
xistinfo <- c("ENSRNOT11111111111", "ENSRNOG11111111111", "Xist")
t2g <- rbind(t2g_ens, xistinfo)
t2g <- dplyr::rename(t2g, target_id = ensembl_transcript_id,
                     ens_gene = ensembl_gene_id, ext_gene = external_gene_name)
```


Differential gene expression whole cells v/s projections

Whole cell samples are either referred to as “CB” (cell body) or “WC” (whole cell) whereas projection samples are referred to as “P”.

The directory structure for file organization is shown below, where cbp\_5rep.txt contains sample information:  
 |-kallisto\_output\_for\_diff\_expression\_by\_sleuth  
  |-cbp\_5rep.txt  
  |-cbp\_5rep  
    |-cbr1  
      |-kallisto  
        |-abundance.tsv  
    |-cbr2  
      |-kallisto  
        |-abundance.tsv  
    …  
    …  
    …  
    |-pr5  
      |-kallisto  
        |-abundance.tsv


```
sample_id_cbp <- dir(file.path(base_dir,"cbp_5rep"))
sample_id_cbp
```


```
 [1] "cbr1" "cbr2" "cbr3" "cbr4" "cbr5" "pr1"  "pr2"  "pr3"  "pr4"  "pr5"
```


```
kal_dirs_cbp <- sapply(sample_id_cbp, function(id) file.path(base_dir, "cbp_5rep", id, "kallisto"))
kal_dirs_cbp
```


```
                                                                                                                                              cbr1 
"../data_tables_for_saini_et_al_19/Intermediate_tables_to_reproduce_figures/kallisto_output_for_diff_expression_by_sleuth//cbp_5rep/cbr1/kallisto" 
                                                                                                                                              cbr2 
"../data_tables_for_saini_et_al_19/Intermediate_tables_to_reproduce_figures/kallisto_output_for_diff_expression_by_sleuth//cbp_5rep/cbr2/kallisto" 
                                                                                                                                              cbr3 
"../data_tables_for_saini_et_al_19/Intermediate_tables_to_reproduce_figures/kallisto_output_for_diff_expression_by_sleuth//cbp_5rep/cbr3/kallisto" 
                                                                                                                                              cbr4 
"../data_tables_for_saini_et_al_19/Intermediate_tables_to_reproduce_figures/kallisto_output_for_diff_expression_by_sleuth//cbp_5rep/cbr4/kallisto" 
                                                                                                                                              cbr5 
"../data_tables_for_saini_et_al_19/Intermediate_tables_to_reproduce_figures/kallisto_output_for_diff_expression_by_sleuth//cbp_5rep/cbr5/kallisto" 
                                                                                                                                               pr1 
 "../data_tables_for_saini_et_al_19/Intermediate_tables_to_reproduce_figures/kallisto_output_for_diff_expression_by_sleuth//cbp_5rep/pr1/kallisto" 
                                                                                                                                               pr2 
 "../data_tables_for_saini_et_al_19/Intermediate_tables_to_reproduce_figures/kallisto_output_for_diff_expression_by_sleuth//cbp_5rep/pr2/kallisto" 
                                                                                                                                               pr3 
 "../data_tables_for_saini_et_al_19/Intermediate_tables_to_reproduce_figures/kallisto_output_for_diff_expression_by_sleuth//cbp_5rep/pr3/kallisto" 
                                                                                                                                               pr4 
 "../data_tables_for_saini_et_al_19/Intermediate_tables_to_reproduce_figures/kallisto_output_for_diff_expression_by_sleuth//cbp_5rep/pr4/kallisto" 
                                                                                                                                               pr5 
 "../data_tables_for_saini_et_al_19/Intermediate_tables_to_reproduce_figures/kallisto_output_for_diff_expression_by_sleuth//cbp_5rep/pr5/kallisto"
```


```
s2c_cbp <- read.table(file.path(base_dir, "cbp_5rep.txt"), header = TRUE, stringsAsFactors=FALSE)
s2c_cbp <- dplyr::select(s2c_cbp, sample = run_accession, condition)
s2c_cbp
```


```
s2c_cbp <- dplyr::mutate(s2c_cbp, path = kal_dirs_cbp)
print(s2c_cbp)
```


```
so_cbp <- sleuth_prep(s2c_cbp, ~ condition, target_mapping = t2g, extra_bootstrap_summary=TRUE)
```


```
It appears that you are running Sleuth from within Rstudio.
Because of concerns with forking processes from a GUI, 'num_cores' is being set to 1.
If you wish to take advantage of multiple cores, please consider running sleuth from the command line.reading in kallisto results
dropping unused factor levels
..........
intersection between target_id from kallisto runs and the target_mapping is empty. attempted to fix problem by removing .N from target_id, then merging back into target_mapping. please check obj$target_mapping to ensure this new mapping is correct.normalizing est_counts
19817 targets passed the filter
normalizing tpm
merging in metadata
summarizing bootstraps
..........
```


```
so_cbp <- sleuth_fit(so_cbp)
```


```
fitting measurement error models
shrinkage estimation
1 NA values were found during variance shrinkage estimation due to mean observation values outside of the range used for the LOESS fit.
The LOESS fit will be repeated using exact computation of the fitted surface to extrapolate the missing values.
These are the target ids with NA values: ENSRNOT00000069133.1
computing variance of betas
```


```
so_cbp <- sleuth_fit(so_cbp, ~1, 'reduced')
```


```
fitting measurement error models
shrinkage estimation
7 NA values were found during variance shrinkage estimation due to mean observation values outside of the range used for the LOESS fit.
The LOESS fit will be repeated using exact computation of the fitted surface to extrapolate the missing values.
These are the target ids with NA values: ENSRNOT00000026089.7, ENSRNOT00000076330.1, ENSRNOT00000076849.1, ENSRNOT00000080526.1, ENSRNOT00000087850.1, ENSRNOT00000092268.1, ENSRNOT00000069133.1
computing variance of betas
```


```
so_cbp <- sleuth_lrt(so_cbp, 'reduced', 'full')
so_cbp <- sleuth_wt(so_cbp, which_beta='conditionP', 'full')
models(so_cbp)
```


```
[  full  ]
formula:  ~condition 
data modeled:  obs_counts 
transform sync'ed:  TRUE 
coefficients:
    (Intercept)
    conditionP
[  reduced  ]
formula:  ~1 
data modeled:  obs_counts 
transform sync'ed:  TRUE 
coefficients:
    (Intercept)
```


```
# Making master data tables  
results_so_lrt_cbp <- sleuth_results(so_cbp, 'reduced:full', test_type = 'lrt')
results_so_wt_cbp <- sleuth_results(so_cbp, 'conditionP', which_model='full', test_type = 'wt')
kal_genes_cbp <- kallisto_table(so_cbp)
so_full_summary_cbp <- so_cbp$fits[['full']]$summary
kal_genes_cbp_wide <- dcast(kal_genes_cbp, target_id ~ sample, value.var="tpm")
colnames(kal_genes_cbp_wide) <- c("target_id", "cbr1_tpm", "cbr2_tpm", "cbr3_tpm", "pr1_tpm", "pr2_tpm", "pr3_tpm", "cbr4_tpm", "pr4_tpm", "cbr5_tpm", "pr5_tpm")
results_so_cbp_wide_kal_tpm <- left_join(results_so_wt_cbp, kal_genes_cbp_wide, by = "target_id")
#write.table(results_so_cbp_wide_kal_tpm, "./sleuth_RNA_diffexp_WC_vs_P.tsv", sep = "\t", col.names=T, row.names=F)  
colnames(results_so_cbp_wide_kal_tpm)
```


```
 [1] "ens_gene"        "ext_gene"        "target_id"       "pval"            "qval"            "b"              
 [7] "se_b"            "mean_obs"        "var_obs"         "tech_var"        "sigma_sq"        "smooth_sigma_sq"
[13] "final_sigma_sq"  "cbr1_tpm"        "cbr2_tpm"        "cbr3_tpm"        "pr1_tpm"         "pr2_tpm"        
[19] "pr3_tpm"         "cbr4_tpm"        "pr4_tpm"         "cbr5_tpm"        "pr5_tpm"
```


Sleuth output is provided in tab separated supplementary table: TableS2\_sleuth\_RNA\_diffexp\_WC\_vs\_P.tsv  
The columns were reorganized for clarity.  
Malat1 is annotated in three parts: AC134224.1 .2 and .3  
In the output file provided, .2 and .3 were deleted .2 and .3, and .1 was renamed to Malat1.

For interactive visualization of differential expression results, launch sleuth\_live:


```
#sleuth_live(so_cbp)
```


```
sessionInfo()
```


```
R version 3.5.1 (2018-07-02)
Platform: x86_64-apple-darwin15.6.0 (64-bit)
Running under: macOS High Sierra 10.13.6

Matrix products: default
BLAS: /System/Library/Frameworks/Accelerate.framework/Versions/A/Frameworks/vecLib.framework/Versions/A/libBLAS.dylib
LAPACK: /Library/Frameworks/R.framework/Versions/3.5/Resources/lib/libRlapack.dylib

locale:
[1] en_US.UTF-8/en_US.UTF-8/en_US.UTF-8/C/en_US.UTF-8/en_US.UTF-8

attached base packages:
[1] stats     graphics  grDevices utils     datasets  methods   base     

other attached packages:
[1] bindrcpp_0.2.2 dplyr_0.7.8    reshape2_1.4.3 sleuth_0.30.0  shiny_1.2.0   

loaded via a namespace (and not attached):
 [1] Rcpp_1.0.0           tidyr_0.8.2          prettyunits_1.0.2    assertthat_0.2.0     digest_0.6.18       
 [6] mime_0.6             R6_2.3.0             plyr_1.8.4           stats4_3.5.1         RSQLite_2.1.1       
[11] evaluate_0.12        httr_1.4.0           ggplot2_3.1.0        pillar_1.3.1         rlang_0.3.1         
[16] progress_1.2.0       lazyeval_0.2.1       curl_3.3             rstudioapi_0.9.0     data.table_1.12.0   
[21] blob_1.1.1           S4Vectors_0.20.1     rmarkdown_1.11       stringr_1.3.1        RCurl_1.95-4.11     
[26] bit_1.1-14           biomaRt_2.38.0       munsell_0.5.0        compiler_3.5.1       httpuv_1.4.5.1      
[31] xfun_0.4             pkgconfig_2.0.2      BiocGenerics_0.28.0  base64enc_0.1-3      htmltools_0.3.6     
[36] tidyselect_0.2.5     tibble_2.0.1         matrixStats_0.54.0   IRanges_2.16.0       XML_3.98-1.16       
[41] crayon_1.3.4         later_0.7.5          bitops_1.0-6         grid_3.5.1           jsonlite_1.6        
[46] xtable_1.8-3         gtable_0.2.0         DBI_1.0.0            magrittr_1.5         scales_1.0.0        
[51] stringi_1.2.4        promises_1.0.1       Rhdf5lib_1.4.2       tools_3.5.1          bit64_0.9-7         
[56] Biobase_2.42.0       glue_1.3.0           purrr_0.3.0          hms_0.4.2            parallel_3.5.1      
[61] yaml_2.2.0           AnnotationDbi_1.44.0 colorspace_1.4-0     rhdf5_2.26.2         memoise_1.1.0       
[66] knitr_1.21           bindr_0.1.1
```

LS0tCnRpdGxlOiAiUk5BIGRpZmZlcmVudGlhbCBleHByZXNzaW9uIGFuYWx5c2lzIGZvciB3aG9sZS1jZWxscyB2L3MgcHJvamVjdGlvbnMiCmF1dGhvcjogSGFybGVlbiBTYWluaQpkYXRlOiBPY3RvYmVyIDE5LCAyMDE5Cm91dHB1dDogaHRtbF9ub3RlYm9vawotLS0KCmBgYHtyfQpsaWJyYXJ5KHNoaW55KQpsaWJyYXJ5KHNsZXV0aCkKbGlicmFyeShyZXNoYXBlMikKbGlicmFyeShkcGx5cikKYGBgCgpgYGB7cn0KIyBzZXR0aW5nIHRoZSBwYXRoIHRvIEthbGxpc3RvIHJlc3VsdHMgaW4gImJhc2VfZGlyIiAgCmJhc2VfZGlyIDwtICIuLi9kYXRhX3RhYmxlc19mb3Jfc2FpbmlfZXRfYWxfMTkva2FsbGlzdG9fb3V0cHV0X2Zvcl9kaWZmX2V4cHJlc3Npb25fYnlfc2xldXRoLyIKCiMgVG8gaW5jbHVkZSBnZW5lIG5hbWVzIGludG8gdHJhbnNjcmlwdC1sZXZlbCBhbmFseXNpcywgcHVsbGluZyBFbnNlbWJsIGRhdGEgdXNpbmcgYmlvbWFSdDogIAptYXJ0IDwtIGJpb21hUnQ6OnVzZU1hcnQoYmlvbWFydCA9ICJFTlNFTUJMX01BUlRfRU5TRU1CTCIsCiAgICAgICAgICAgICAgICAgICAgICAgICBkYXRhc2V0ID0gInJub3J2ZWdpY3VzX2dlbmVfZW5zZW1ibCIsCiAgICAgICAgICAgICAgICAgICAgICAgICBob3N0ID0gInVzZWFzdC5lbnNlbWJsLm9yZyIpCgp0MmdfZW5zIDwtIGJpb21hUnQ6OmdldEJNKGF0dHJpYnV0ZXMgPSBjKCJlbnNlbWJsX3RyYW5zY3JpcHRfaWQiLCAiZW5zZW1ibF9nZW5lX2lkIiwKICAgICAgICAgICAgICAgICAgICAgICAgICAgICAgICAgICAgICAgICAiZXh0ZXJuYWxfZ2VuZV9uYW1lIiksIG1hcnQgPSBtYXJ0KQoKIyBYaXN0IGlzIG5vdCBhbm5vdGF0ZWQgaW4gdGhlIHJhdCBybm9yNiBnZW5vbWUuICAKIyBVc2VkIExpZnRPdmVyIChVQ1NDL0tlbnQgVG9vbHMpIHRvIGdldCByYXQgWGlzdCBzZXF1ZW5jZSBmb3IgaW5wdXQgdG8gS2FsbGlzdG8gIAojIEFzc2lnbmVkIGFydGlmaWNhbCBlbnNlbWJsIElEcyBmb3IgdGhlIGdlbmUgYW5kIHRyYW5zY3JpcHQgdG8gZW50ZXIgaW50byBTbGV1dGggIAp4aXN0aW5mbyA8LSBjKCJFTlNSTk9UMTExMTExMTExMTEiLCAiRU5TUk5PRzExMTExMTExMTExIiwgIlhpc3QiKQp0MmcgPC0gcmJpbmQodDJnX2VucywgeGlzdGluZm8pCgp0MmcgPC0gZHBseXI6OnJlbmFtZSh0MmcsIHRhcmdldF9pZCA9IGVuc2VtYmxfdHJhbnNjcmlwdF9pZCwKICAgICAgICAgICAgICAgICAgICAgZW5zX2dlbmUgPSBlbnNlbWJsX2dlbmVfaWQsIGV4dF9nZW5lID0gZXh0ZXJuYWxfZ2VuZV9uYW1lKQpgYGAKCiMjIyMjIyMjIyMjIERpZmZlcmVudGlhbCBnZW5lIGV4cHJlc3Npb24gd2hvbGUgY2VsbHMgdi9zIHByb2plY3Rpb25zICMjIyMjIyMjIyMjIApXaG9sZSBjZWxsIHNhbXBsZXMgYXJlIGVpdGhlciByZWZlcnJlZCB0byBhcyAiQ0IiIChjZWxsIGJvZHkpIG9yICJXQyIgKHdob2xlIGNlbGwpIHdoZXJlYXMgcHJvamVjdGlvbiBzYW1wbGVzIGFyZSByZWZlcnJlZCB0byBhcyAiUCIuICAKClRoZSBkaXJlY3Rvcnkgc3RydWN0dXJlIGZvciBmaWxlIG9yZ2FuaXphdGlvbiBpcyBzaG93biBiZWxvdywgd2hlcmUgY2JwXzVyZXAudHh0IGNvbnRhaW5zIHNhbXBsZSBpbmZvcm1hdGlvbjogIAombmJzcDt8LWthbGxpc3RvX291dHB1dF9mb3JfZGlmZl9leHByZXNzaW9uX2J5X3NsZXV0aCAgCiZuYnNwOyZuYnNwO3wtY2JwXzVyZXAudHh0ICAKJm5ic3A7Jm5ic3A7fC1jYnBfNXJlcCAgCiZuYnNwOyZuYnNwOyZuYnNwOyZuYnNwO3wtY2JyMSAgCiZuYnNwOyZuYnNwOyZuYnNwOyZuYnNwOyZuYnNwOyZuYnNwO3wta2FsbGlzdG8gIAombmJzcDsmbmJzcDsmbmJzcDsmbmJzcDsmbmJzcDsmbmJzcDsmbmJzcDsmbmJzcDt8LWFidW5kYW5jZS50c3YgIAombmJzcDsmbmJzcDsmbmJzcDsmbmJzcDt8LWNicjIgIAombmJzcDsmbmJzcDsmbmJzcDsmbmJzcDsmbmJzcDsmbmJzcDt8LWthbGxpc3RvICAKJm5ic3A7Jm5ic3A7Jm5ic3A7Jm5ic3A7Jm5ic3A7Jm5ic3A7Jm5ic3A7Jm5ic3A7fC1hYnVuZGFuY2UudHN2ICAKJm5ic3A7Jm5ic3A7Jm5ic3A7Jm5ic3A7Li4uICAKJm5ic3A7Jm5ic3A7Jm5ic3A7Jm5ic3A7Li4uICAKJm5ic3A7Jm5ic3A7Jm5ic3A7Jm5ic3A7Li4uICAKJm5ic3A7Jm5ic3A7Jm5ic3A7Jm5ic3A7fC1wcjUgIAombmJzcDsmbmJzcDsmbmJzcDsmbmJzcDsmbmJzcDsmbmJzcDt8LWthbGxpc3RvICAKJm5ic3A7Jm5ic3A7Jm5ic3A7Jm5ic3A7Jm5ic3A7Jm5ic3A7Jm5ic3A7Jm5ic3A7fC1hYnVuZGFuY2UudHN2ICAKCmBgYHtyfQpzYW1wbGVfaWRfY2JwIDwtIGRpcihmaWxlLnBhdGgoYmFzZV9kaXIsImNicF81cmVwIikpCnNhbXBsZV9pZF9jYnAKYGBgCgpgYGB7cn0Ka2FsX2RpcnNfY2JwIDwtIHNhcHBseShzYW1wbGVfaWRfY2JwLCBmdW5jdGlvbihpZCkgZmlsZS5wYXRoKGJhc2VfZGlyLCAiY2JwXzVyZXAiLCBpZCwgImthbGxpc3RvIikpCmthbF9kaXJzX2NicApgYGAKCmBgYHtyfQpzMmNfY2JwIDwtIHJlYWQudGFibGUoZmlsZS5wYXRoKGJhc2VfZGlyLCAiY2JwXzVyZXAudHh0IiksIGhlYWRlciA9IFRSVUUsIHN0cmluZ3NBc0ZhY3RvcnM9RkFMU0UpCnMyY19jYnAgPC0gZHBseXI6OnNlbGVjdChzMmNfY2JwLCBzYW1wbGUgPSBydW5fYWNjZXNzaW9uLCBjb25kaXRpb24pCnMyY19jYnAKYGBgCgpgYGB7cn0KczJjX2NicCA8LSBkcGx5cjo6bXV0YXRlKHMyY19jYnAsIHBhdGggPSBrYWxfZGlyc19jYnApCnByaW50KHMyY19jYnApCgpgYGAKYGBge3J9CnNvX2NicCA8LSBzbGV1dGhfcHJlcChzMmNfY2JwLCB+IGNvbmRpdGlvbiwgdGFyZ2V0X21hcHBpbmcgPSB0MmcsIGV4dHJhX2Jvb3RzdHJhcF9zdW1tYXJ5PVRSVUUpCnNvX2NicCA8LSBzbGV1dGhfZml0KHNvX2NicCkKc29fY2JwIDwtIHNsZXV0aF9maXQoc29fY2JwLCB+MSwgJ3JlZHVjZWQnKQpzb19jYnAgPC0gc2xldXRoX2xydChzb19jYnAsICdyZWR1Y2VkJywgJ2Z1bGwnKQpzb19jYnAgPC0gc2xldXRoX3d0KHNvX2NicCwgd2hpY2hfYmV0YT0nY29uZGl0aW9uUCcsICdmdWxsJykKCm1vZGVscyhzb19jYnApCmBgYAoKYGBge3J9CiMgTWFraW5nIG1hc3RlciBkYXRhIHRhYmxlcyAgCnJlc3VsdHNfc29fbHJ0X2NicCA8LSBzbGV1dGhfcmVzdWx0cyhzb19jYnAsICdyZWR1Y2VkOmZ1bGwnLCB0ZXN0X3R5cGUgPSAnbHJ0JykKcmVzdWx0c19zb193dF9jYnAgPC0gc2xldXRoX3Jlc3VsdHMoc29fY2JwLCAnY29uZGl0aW9uUCcsIHdoaWNoX21vZGVsPSdmdWxsJywgdGVzdF90eXBlID0gJ3d0JykKa2FsX2dlbmVzX2NicCA8LSBrYWxsaXN0b190YWJsZShzb19jYnApCnNvX2Z1bGxfc3VtbWFyeV9jYnAgPC0gc29fY2JwJGZpdHNbWydmdWxsJ11dJHN1bW1hcnkKCmthbF9nZW5lc19jYnBfd2lkZSA8LSBkY2FzdChrYWxfZ2VuZXNfY2JwLCB0YXJnZXRfaWQgfiBzYW1wbGUsIHZhbHVlLnZhcj0idHBtIikKY29sbmFtZXMoa2FsX2dlbmVzX2NicF93aWRlKSA8LSBjKCJ0YXJnZXRfaWQiLCAiY2JyMV90cG0iLCAiY2JyMl90cG0iLCAiY2JyM190cG0iLCAicHIxX3RwbSIsICJwcjJfdHBtIiwgInByM190cG0iLCAiY2JyNF90cG0iLCAicHI0X3RwbSIsICJjYnI1X3RwbSIsICJwcjVfdHBtIikKcmVzdWx0c19zb19jYnBfd2lkZV9rYWxfdHBtIDwtIGxlZnRfam9pbihyZXN1bHRzX3NvX3d0X2NicCwga2FsX2dlbmVzX2NicF93aWRlLCBieSA9ICJ0YXJnZXRfaWQiKQoKI3dyaXRlLnRhYmxlKHJlc3VsdHNfc29fY2JwX3dpZGVfa2FsX3RwbSwgIi4vc2xldXRoX1JOQV9kaWZmZXhwX1dDX3ZzX1AudHN2Iiwgc2VwID0gIlx0IiwgY29sLm5hbWVzPVQsIHJvdy5uYW1lcz1GKSAgCgpjb2xuYW1lcyhyZXN1bHRzX3NvX2NicF93aWRlX2thbF90cG0pCmBgYAoKU2xldXRoIG91dHB1dCBpcyBwcm92aWRlZCBpbiB0YWIgc2VwYXJhdGVkIHN1cHBsZW1lbnRhcnkgdGFibGU6IFRhYmxlUzJfc2xldXRoX1JOQV9kaWZmZXhwX1dDX3ZzX1AudHN2ICAKVGhlIGNvbHVtbnMgd2VyZSByZW9yZ2FuaXplZCBmb3IgY2xhcml0eS4gIApNYWxhdDEgaXMgYW5ub3RhdGVkIGluIHRocmVlIHBhcnRzOiBBQzEzNDIyNC4xIC4yIGFuZCAuMyAgCkluIHRoZSBvdXRwdXQgZmlsZSBwcm92aWRlZCwgLjIgYW5kIC4zIHdlcmUgZGVsZXRlZCAuMiBhbmQgLjMsIGFuZCAuMSB3YXMgcmVuYW1lZCB0byBNYWxhdDEuICAKICAKRm9yIGludGVyYWN0aXZlIHZpc3VhbGl6YXRpb24gb2YgZGlmZmVyZW50aWFsIGV4cHJlc3Npb24gcmVzdWx0cywgbGF1bmNoIHNsZXV0aF9saXZlOiAgCgpgYGB7cn0Kc2xldXRoX2xpdmUoc29fY2JwKQpgYGAKCmBgYHtyfQpzZXNzaW9uSW5mbygpCmBgYA==
